# Supplementary material for: A new molecular classification to drive precision treatment strategies in primary Sjögren’s syndrome
Source: Nat Commun. 2021 Jun 10;12:3523. doi: 10.1038/s41467-021-23472-7 (PMC8192578; doi:10.1038/s41467-021-23472-7)
Supplement: Supplementary file 1 — Supplementary Informations [file 41467_2021_23472_MOESM1_ESM.pdf]

Supplementary Figures

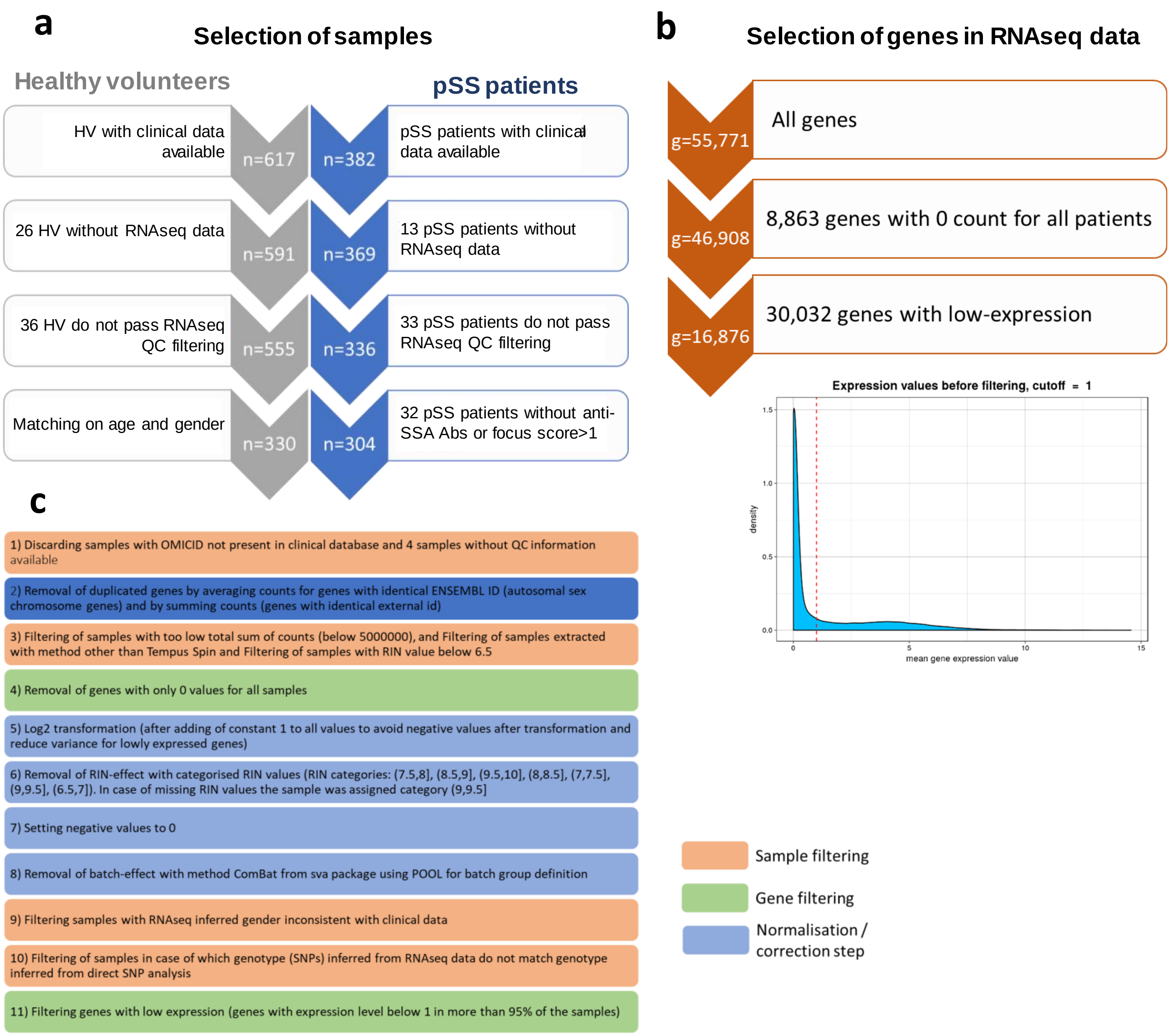

Supplementary Fig. 1: RNAseq analysis flowchart. (**a**) Flow-chart summarizing patient and healthy volunteers (HV) selection in the study. Patients and HV were selected based on the availability of clinical data. Within these patients, the ones without RNAseq data and the ones who do not pass RNAseq quality control (QC) filtering were excluded. (**b**) Flowchart summarizing genes selection. Genes with no counts or low expression were excluded. (c) Flowchart summarizing RNASeq data normalization. Duplicated genes and genes that do not pass QC were excluded. Log2 transformation were applied on gene expression data followed by removal of RNA integrity number (RIN) and batch effect and genes with low expression.

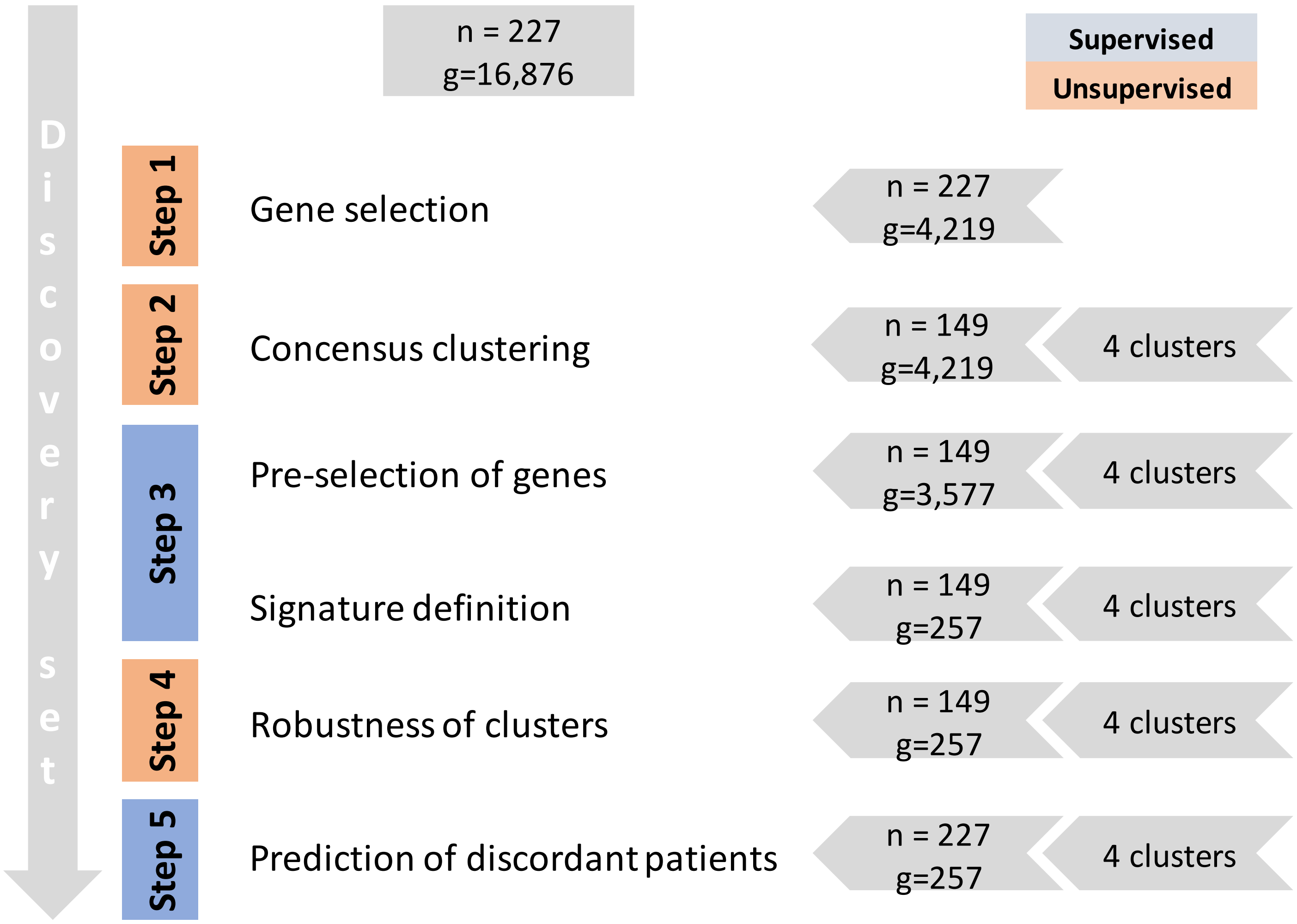

Supplementary Fig. 2: Flow chart of unsupervised hierarchical clustering using genome wide transcriptome data. Primary Sjögren's syndrome (pSS) patients were classified with 3 unsupervised methods in 4 clusters. Out of the 227 patients, 149 yielded a consensus subgroup assignment in a cluster. Supervised classification identified a minimal list of 257 discriminative genes signature. A centroid based predictor was used to predict discordant patients.

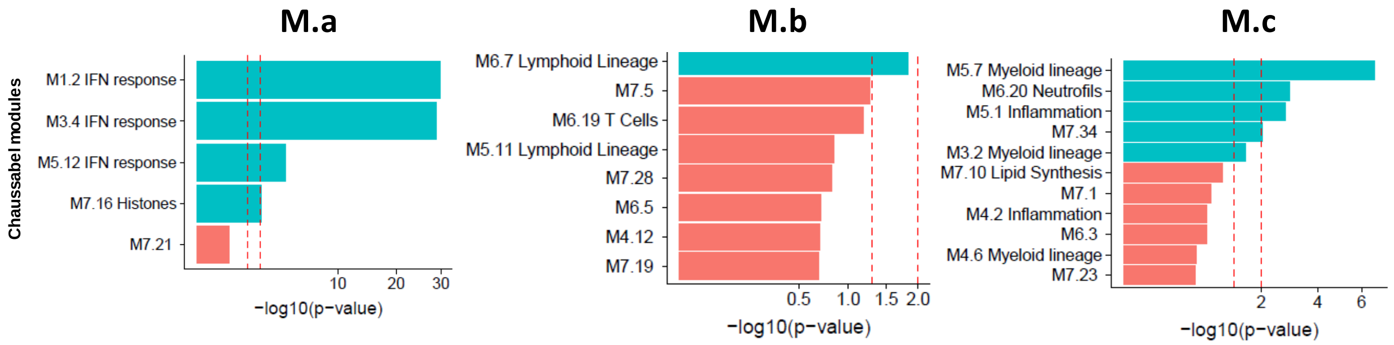

Supplementary Fig. 3: Enrichment of blood transcriptional modules. Box plots represent the enrichment of each genes modules from the random forest signature using blood modules as gene sets and expressed as -log10(p-value) [1]. The random forest signature is divided into 3 modules: M.a (105 genes), M.b (20 genes) and M.c (132 genes). Statistical significance was determined by Fisher's Exact test and gene modules with a p-value <0.05 were considered significant. The blue bar indicates significant modules and the red bars non-significant modules. Red dotted lines correspond to significant threshold (0.05 and 0.01).

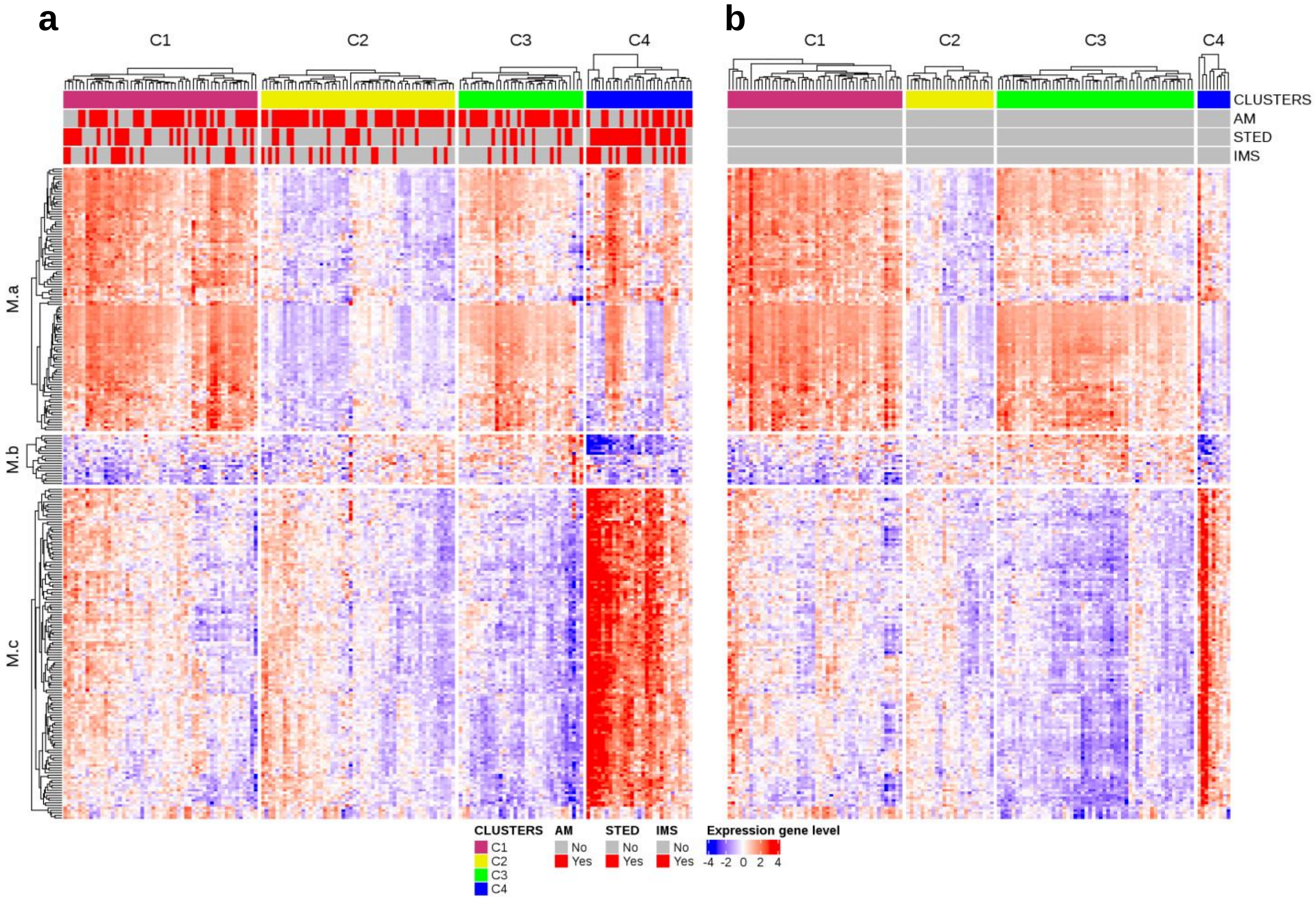

Supplementary Fig. 4: Impact of treatments on cluster distribution. Heatmap performed on 304 pSS patients shows the distribution of gene transcripts across the 4 clusters in 169 treated (**a**) versus 135 untreated (**b**) patients. In columns patients are grouped by cluster assignment and in rows the genes are grouped by functional assignment modules (RF genes modules). Red represents genes over-expression and blue represents genes under-expression. At the top of the figure, annotations show each of the treatment groups for each individual (AM: antimalarials, STED: steroids, IMS: immunosuppressors, red represents patient with treatment and grey represents patient without treatment).

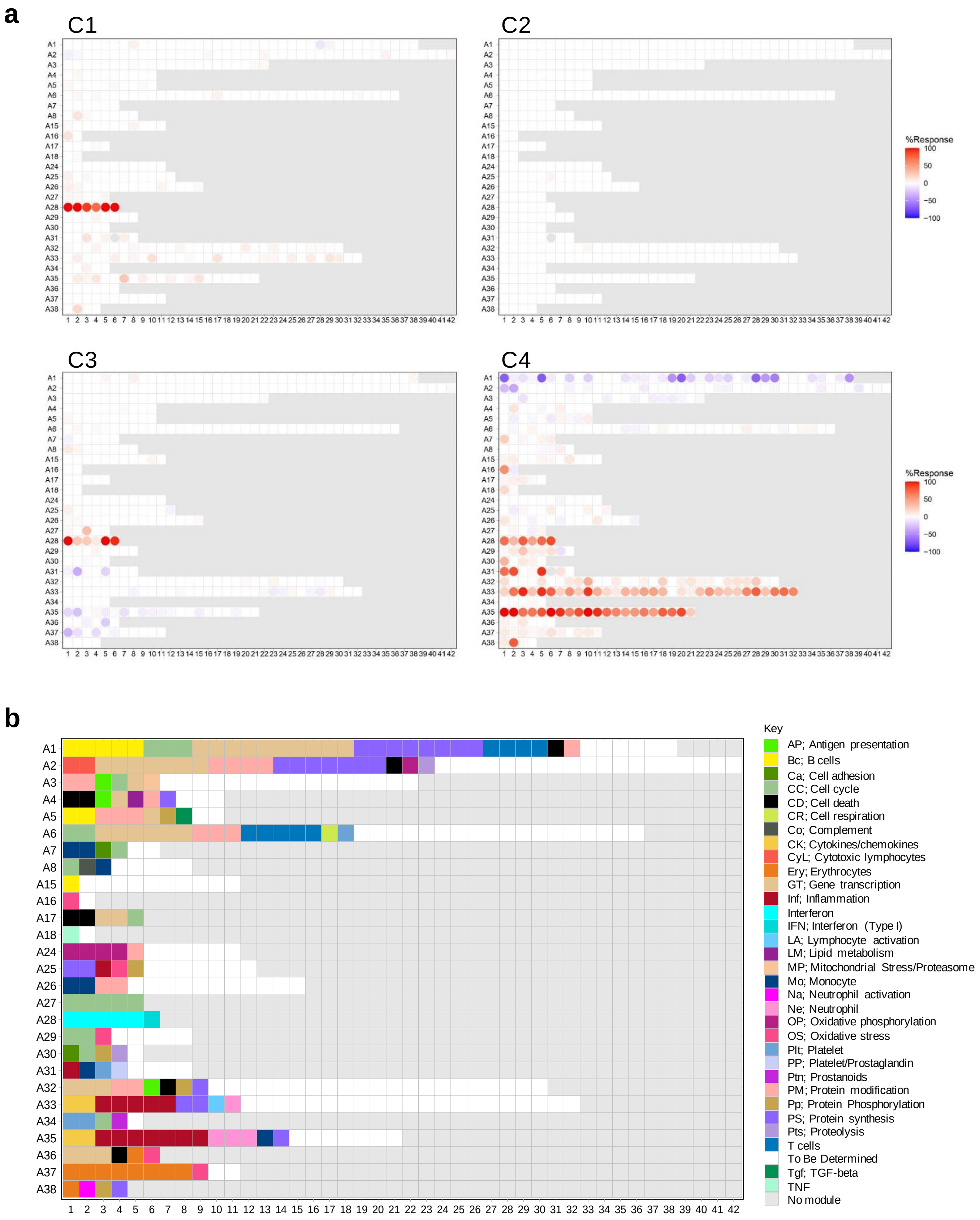

Supplementary Fig. 5: Fingerprint grid plots mapping transcriptome repertoire perturbations across the four pSS clusters. Rinchai et al. [2] have developed a R package, BloodGen3Module which allowed group comparison analyses to be performed at a module-level, and to display the results as annotated fingerprint grid plots. A set of 382 modules covering 14,168 transcripts were defined on the basis of co-clustering observed across 16 different states, encompassing autoimmune and infectious diseases, primary immune deficiency, cancer and pregnancy, representing 985 unique transcriptome profiles [3]. From these 382 modules (genes sets), a reduced level with 38 variables was built (A1 to A38) constituted by modules sets and functionally annotated pathway, ontology and literature term enrichment. (**a**) Changes in blood transcript abundance in subjects from each cluster compared to healthy volunteers (HV) with a fold change cut-off = 1.5 and a false discovery rate adjusted p-value < 0.05 are represented on the fingerprint

grid plot. The modules occupy a fixed position on the fingerprint grid plots. An increase in transcript abundance for a given module is represented by a red spot; a decrease in abundance is represented by a blue spot. Modules arranged on a given row belong to a module aggregate (here denoted as A1 to A38). Changes measured at the "aggregate-level" are represented by spots to the left of the grid next to the denomination for the corresponding aggregate. The colors and intensities of the spots are based on the average across each given row of modules. (**b**) A module annotation grid was provided by BloodGen3Module R package [2] (https://github.com/Drinchai/BloodGen3Module) where a color key indicates the functional associations attributed to some of the modules on the grid. Positions on the annotation grid occupied by modules for which no consensus annotation was attributed are colored white. Positions on the grid for which no modules have been assigned are colored grey.

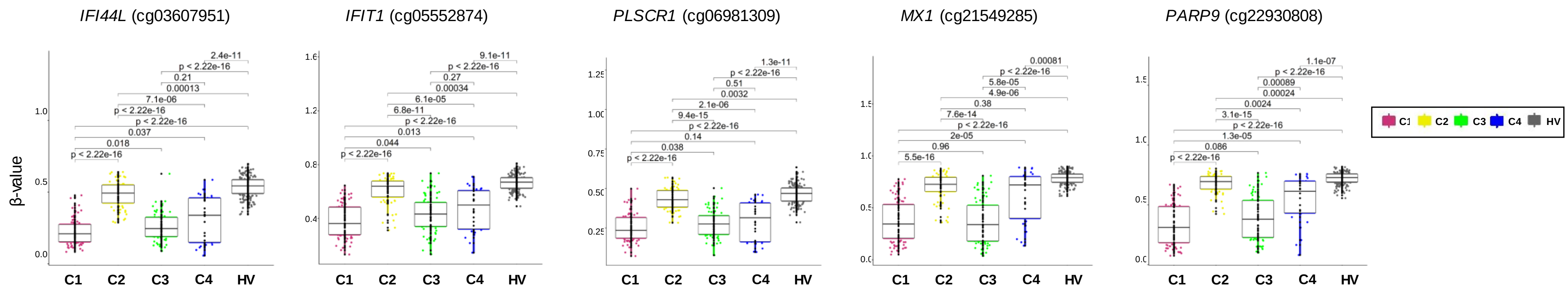

Supplementary Fig. 6: Methylation level analysis of the 5 CpG common to C1, C2 and C3 between 226 patients (C1: 81, C2: 57, C3: 62 and C4: 26) and 175 healthy volunteers (HV). Statistical significance was determined by two-tailed pairwise Wilcoxon-rank sum test. Plots show median with error bars indicating ± interquartile range (IQR).

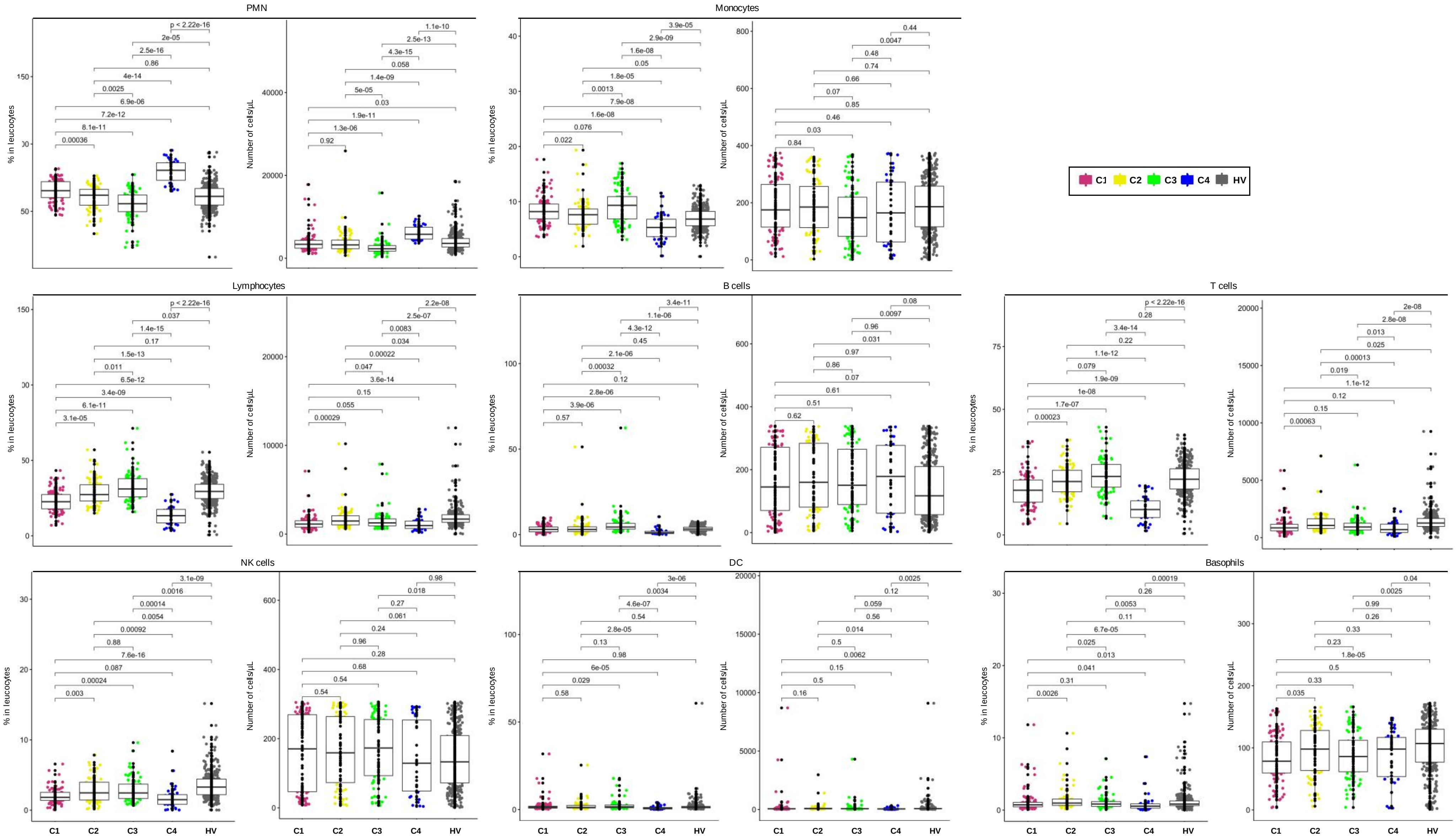
PMN
Monocytes
Lymphocytes
B cells
T cells
NK cells
DC
Basophils
% in leucocytes
Number of cells/µL
C1
C2
C3
C4
HV

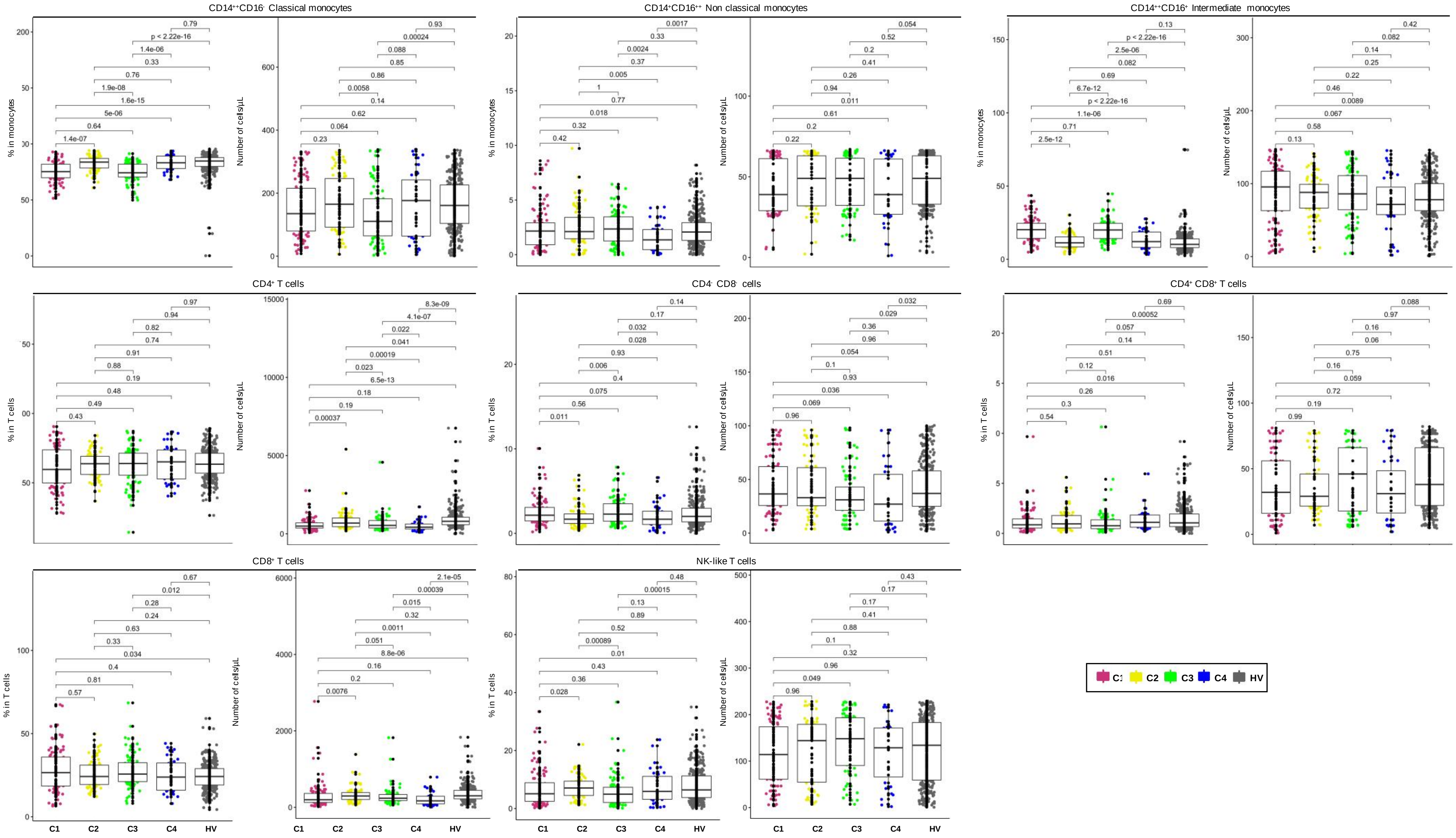

CD14++CD16- Classical monocytes
CD14+CD16++ Non classical monocytes
CD14++CD16+ Intermediate monocytes
CD4+ T cells
CD4- CD8- cells
CD4+ CD8+ T cells
CD8+ T cells
NK-like T cells
% in monocytes
% in T cells
Number of cells/µL
C1
C2
C3
C4
HV

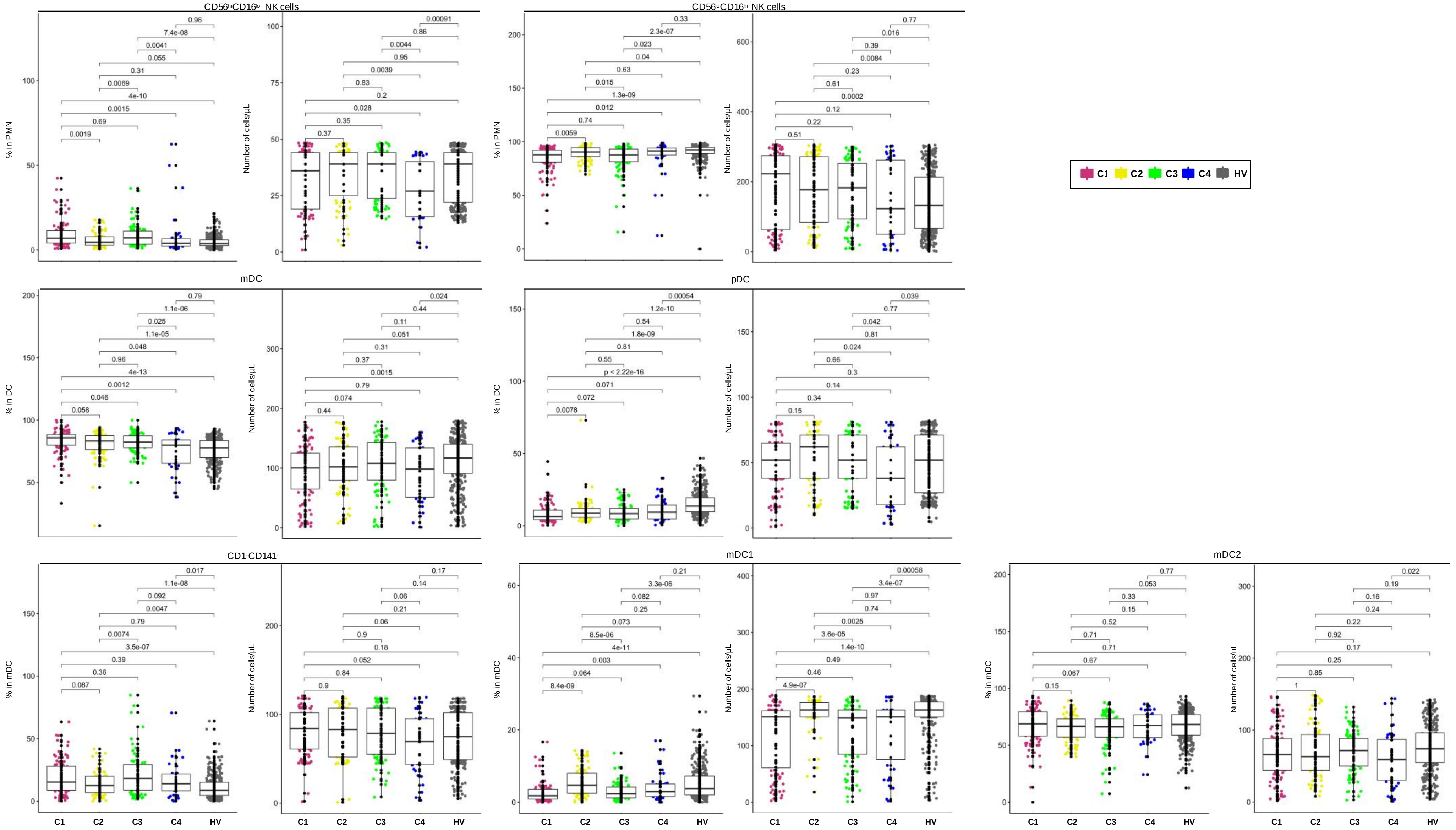

CD56hiCD16lo NK cells
% in PMN
Number of cells/µL
0.96
7.4e-08
0.0041
0.055
0.31
0.0069
4e-10
0.0015
0.69
0.0019
0.00091
0.86
0.0044
0.95
0.0039
0.83
0.2
0.028
0.35
0.37
CD56loCD16hi NK cells
0.33
2.3e-07
0.023
0.04
0.63
0.015
1.3e-09
0.012
0.74
0.0059
0.77
0.016
0.39
0.0084
0.23
0.61
0.0002
0.12
0.22
0.51
C1
C2
C3
C4
HV
mDC
% in DC
0.79
1.1e-06
0.025
1.1e-05
0.048
0.96
4e-13
0.0012
0.046
0.058
0.024
0.44
0.11
0.051
0.31
0.37
0.0015
0.79
0.074
0.44
pDC
0.00054
1.2e-10
0.54
1.8e-09
0.81
0.55
p < 2.22e-16
0.071
0.072
0.0078
0.039
0.77
0.042
0.81
0.024
0.66
0.3
0.14
0.34
0.15
CD1-CD141-
% in mDC
0.017
1.1e-08
0.092
0.0047
0.79
0.0074
3.5e-07
0.39
0.36
0.087
0.17
0.14
0.06
0.21
0.06
0.9
0.18
0.052
0.84
0.9
mDC1
0.21
3.3e-06
0.082
0.25
0.073
8.5e-06
4e-11
0.003
0.064
8.4e-09
0.00058
3.4e-07
0.97
0.74
0.0025
3.6e-05
1.4e-10
0.49
0.46
4.9e-07
mDC2
0.77
0.053
0.33
0.15
0.52
0.71
0.71
0.67
0.067
0.15
0.022
0.19
0.16
0.24
0.22
0.92
0.17
0.25
0.85
1

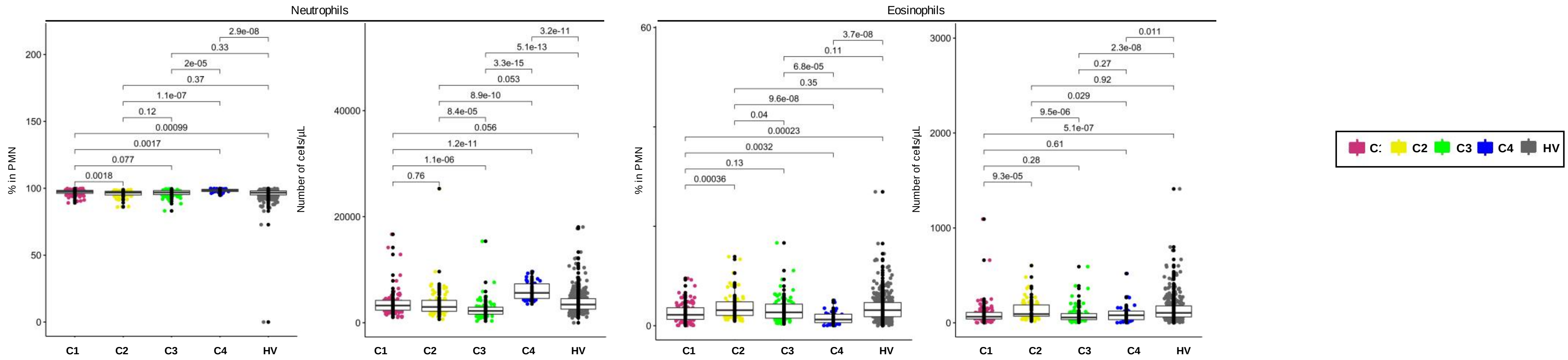

Supplementary Fig. 7: Blood cell subsets distribution between clusters and healthy volunteers (HV). Cell subset distribution was assessed by Flow cytometry for 283 patients (C1: 96, C2: 71, C3: 80 and C4: 36) and 309 HV. For a given cell subset, the graph on the left represents the proportion (percentage) of the subset in the defined population (look at the legend on the y-axis) and the graph on the right represents the absolute count expressed in number of cells/µL in total blood. The gating strategy is detailed in [4] and in supplementary Fig.15. Statistical significance was determined by two-tailed pairwise Wilcoxon-rank sum test. Plots show median with error bars indicating ± interquartile range (IQR).

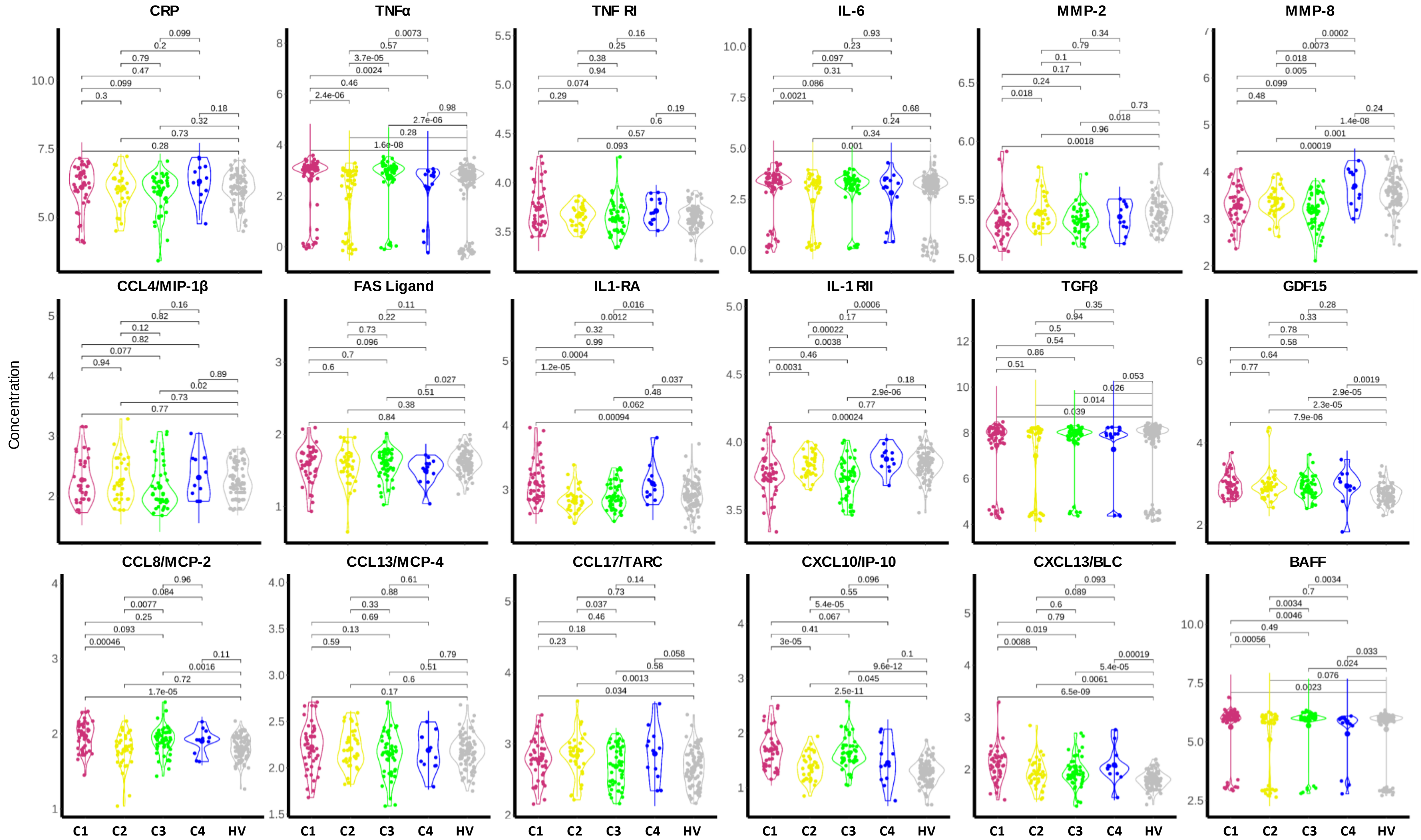

Supplementary Fig. 8: Boxplots representing the cytokine distribution in the 4 clusters of pSS patients and in healthy volunteers (HV). Soluble mediators were analyzed in 192 pSS patients (C1: 67, C2: 48, C3: 61 and C4: 16) and 171 HV. CXCL13/BLC, FAS Ligand, GDF-15, CXCL10/IP-10, CCL8/MCP-2, CCL13/MCP-4, CCL4/MIP-1β, MMP-8, CCL17/TARC, IL-1 RII, TNF RI and IL1-RA were measured using the Luminex system and expressed as pg/ml. Soluble MMP-2, CRP, TNFα, IL-6, BAFF and TGFβ were measured by the quantitative sandwich

enzyme immunoassay technique and expressed as pg/ml. The differential cytokine concentration comparisons between clusters and HV were performed using two-tailed pairwise Wilcoxon-rank sum test and expressed as p-value. Error bars represent the standard deviation.

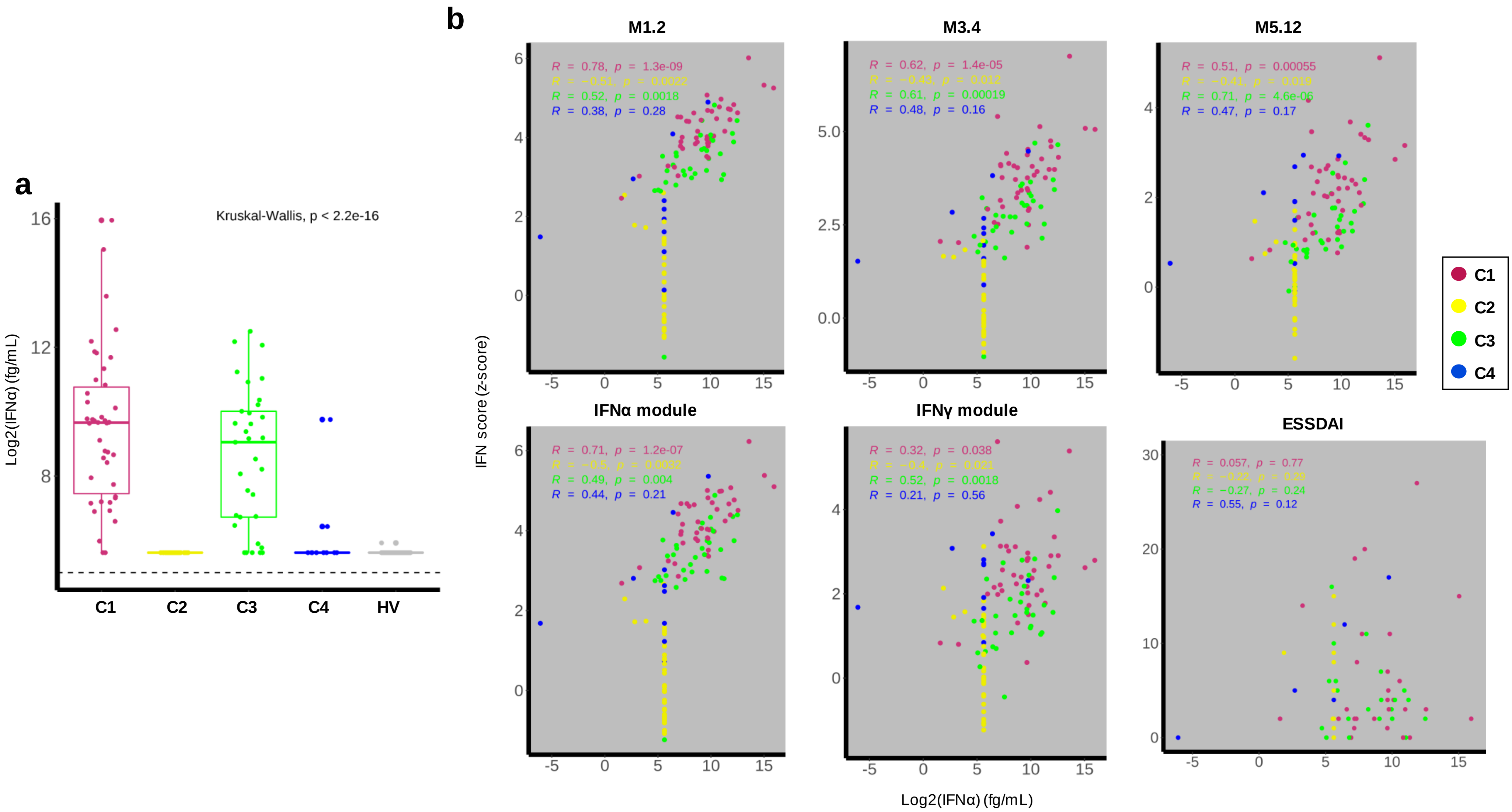

Supplementary Fig. 9: Simoa quantification of IFNα in serum and correlations with IFN z-scores and ESSDAI. Serum levels of IFNα were assessed in 115 pSS patients (C1: 39, C2: 35, C3:32 and C4: 9) and 118 healthy volunteers (HV). (**a**) Boxplot represents the distribution of log2 IFNα concentration quantified by Simoa and expressed in fg/mL in the various pSS clusters and in HV. The concentration was compared with two-sided Kruskal-Wallis test. Plots show median with error bars indicating ± interquartile range. (**b**) Scatterplots show the correlation of Pearson between the log2 IFNα concentration and IFN z-scores or ESSDAI per cluster. Associated p-value from Pearson correlation test for each cluster is shown. M1.2, M3.4, M5.12 IFN blood module [5] and IFNα and IFNγ modules [6] were used to calculate IFN scores.

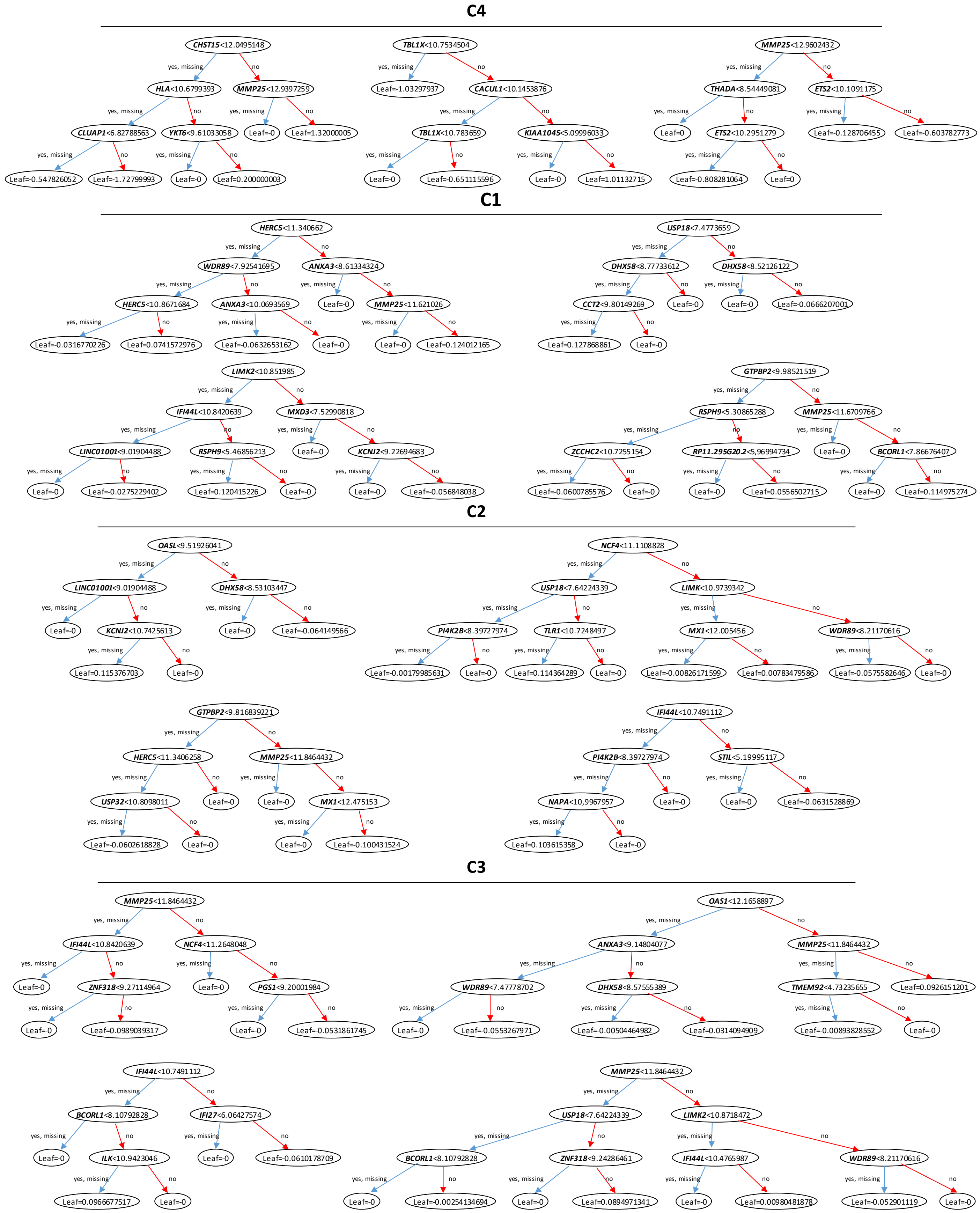

Supplementary Fig. 10: Meta model composition. Detailed composition of each tree (predictor) forming the two models (C4 and C1-C2-C3). For the C4 model, 3 predictors were used to discriminate between C4 versus all, for the C1-C2-C3 model, 4 predictors were used to predict each cluster. For both models, the maximum depth of the predictors is set at 3. Altogether, the model is made up of 15 predictors. Each predictor is composed of nodes evaluating specific parameters of an observation and leaves containing scores corresponding to the probability of the observation belonging to the class evaluated by the predictor subset (in our case C1, C2, C3 and C4). The final probability is the sum of the leaf values transformed by a sigmoid function. Leaf values can be negative, with a score of 0 representing a probability of 50%.

a

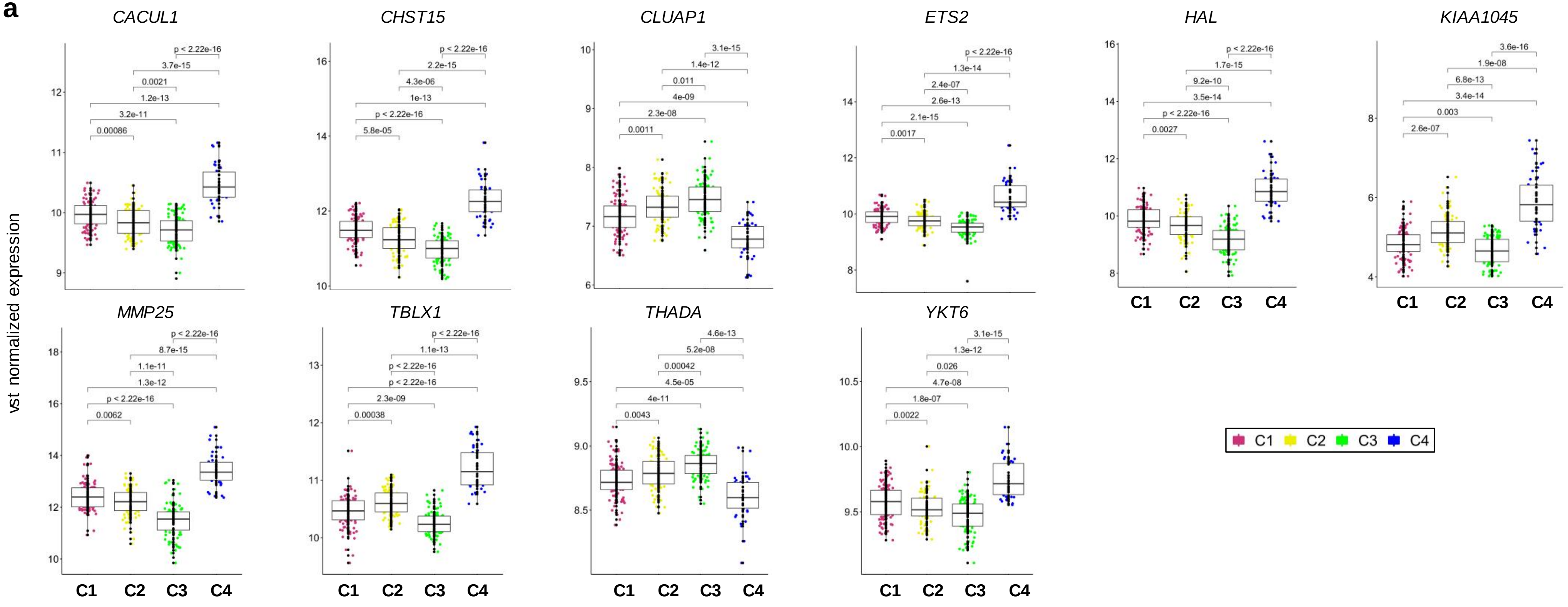
vst normalized expression
CACUL1
p < 2.22e-16
3.7e-15
0.0021
1.2e-13
3.2e-11
0.00086
CHST15
p < 2.22e-16
2.2e-15
4.3e-06
1e-13
p < 2.22e-16
5.8e-05
CLUAP1
3.1e-15
1.4e-12
0.011
4e-09
2.3e-08
0.0011
ETS2
p < 2.22e-16
1.3e-14
2.4e-07
2.6e-13
2.1e-15
0.0017
HAL
p < 2.22e-16
1.7e-15
9.2e-10
3.5e-14
p < 2.22e-16
0.0027
KIAA1045
3.6e-16
1.9e-08
6.8e-13
3.4e-14
0.003
2.6e-07
MMP25
p < 2.22e-16
8.7e-15
1.1e-11
1.3e-12
p < 2.22e-16
0.0062
TBLX1
p < 2.22e-16
1.1e-13
p < 2.22e-16
p < 2.22e-16
2.3e-09
0.00038
THADA
4.6e-13
5.2e-08
0.00042
4.5e-05
4e-11
0.0043
YKT6
3.1e-15
1.3e-12
0.026
4.7e-08
1.8e-07
0.0022
C1 C2 C3 C4
C1 C2 C3 C4

b

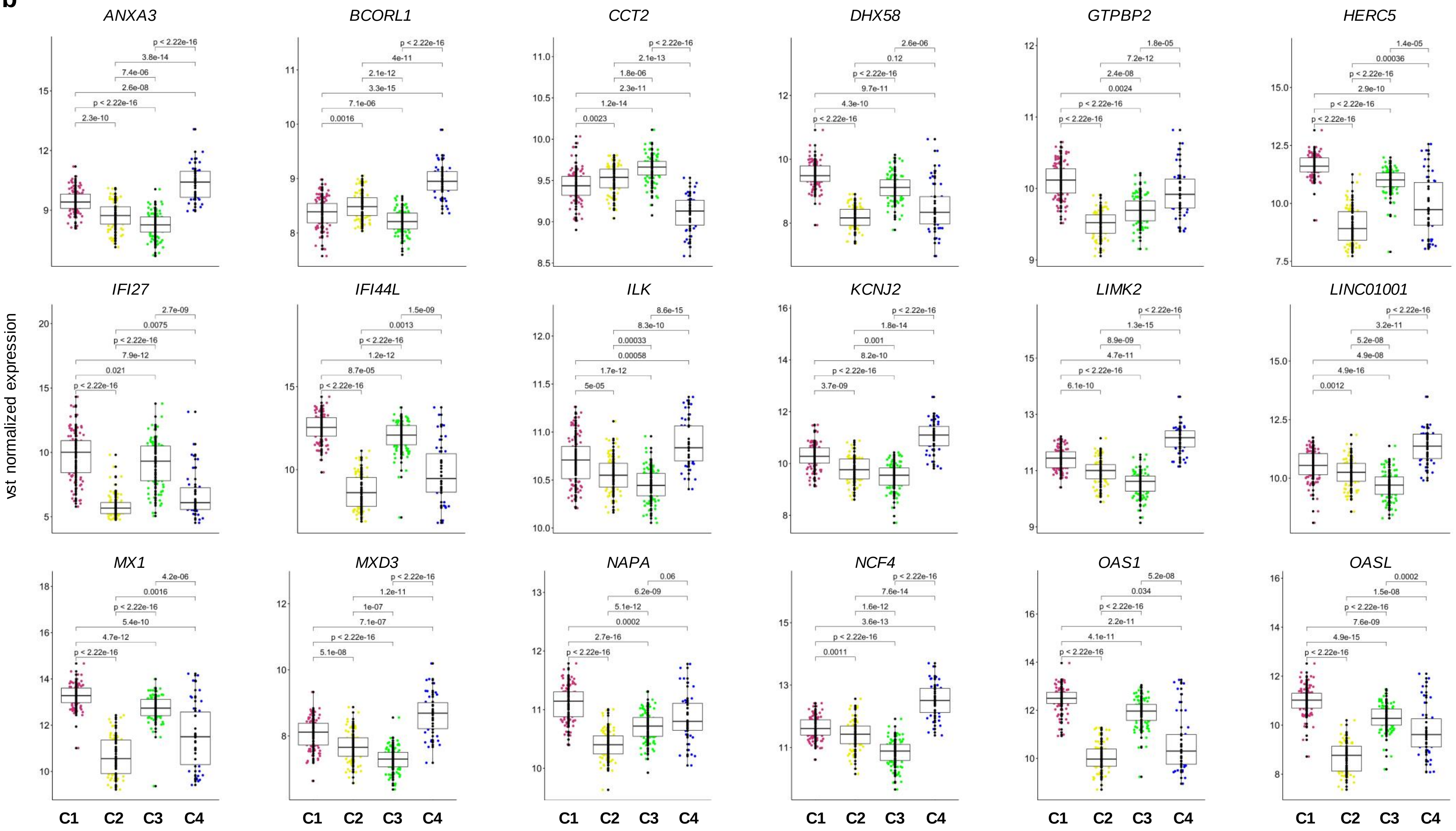
vst normalized expression
ANXA3
p < 2.22e-16
3.8e-14
7.4e-06
2.6e-08
p < 2.22e-16
2.3e-10
BCORL1
p < 2.22e-16
4e-11
2.1e-12
3.3e-15
7.1e-06
0.0016
CCT2
p < 2.22e-16
2.1e-13
1.8e-06
2.3e-11
1.2e-14
0.0023
DHX58
2.6e-06
0.12
p < 2.22e-16
9.7e-11
4.3e-10
p < 2.22e-16
GTPBP2
1.8e-05
7.2e-12
2.4e-08
0.0024
p < 2.22e-16
p < 2.22e-16
HERC5
1.4e-05
0.00036
p < 2.22e-16
2.9e-10
p < 2.22e-16
p < 2.22e-16
IFI27
2.7e-09
0.0075
p < 2.22e-16
7.9e-12
0.021
p < 2.22e-16
IFI44L
1.5e-09
0.0013
p < 2.22e-16
1.2e-12
8.7e-05
p < 2.22e-16
ILK
8.6e-15
8.3e-10
0.00033
0.00058
1.7e-12
5e-05
KCNJ2
p < 2.22e-16
1.8e-14
0.001
8.2e-10
p < 2.22e-16
3.7e-09
LIMK2
p < 2.22e-16
1.3e-15
8.9e-09
4.7e-11
p < 2.22e-16
6.1e-10
LINC01001
p < 2.22e-16
3.2e-11
5.2e-08
4.9e-08
4.9e-16
0.0012
MX1
4.2e-06
0.0016
p < 2.22e-16
5.4e-10
4.7e-12
p < 2.22e-16
MXD3
p < 2.22e-16
1.2e-11
1e-07
7.1e-07
p < 2.22e-16
5.1e-08
NAPA
0.06
6.2e-09
5.1e-12
0.0002
2.7e-16
p < 2.22e-16
NCF4
p < 2.22e-16
7.6e-14
1.6e-12
3.6e-13
p < 2.22e-16
0.0011
OAS1
5.2e-08
0.034
p < 2.22e-16
2.2e-11
4.1e-11
p < 2.22e-16
OASL
0.0002
1.5e-08
p < 2.22e-16
7.6e-09
4.9e-15
p < 2.22e-16
C1 C2 C3 C4

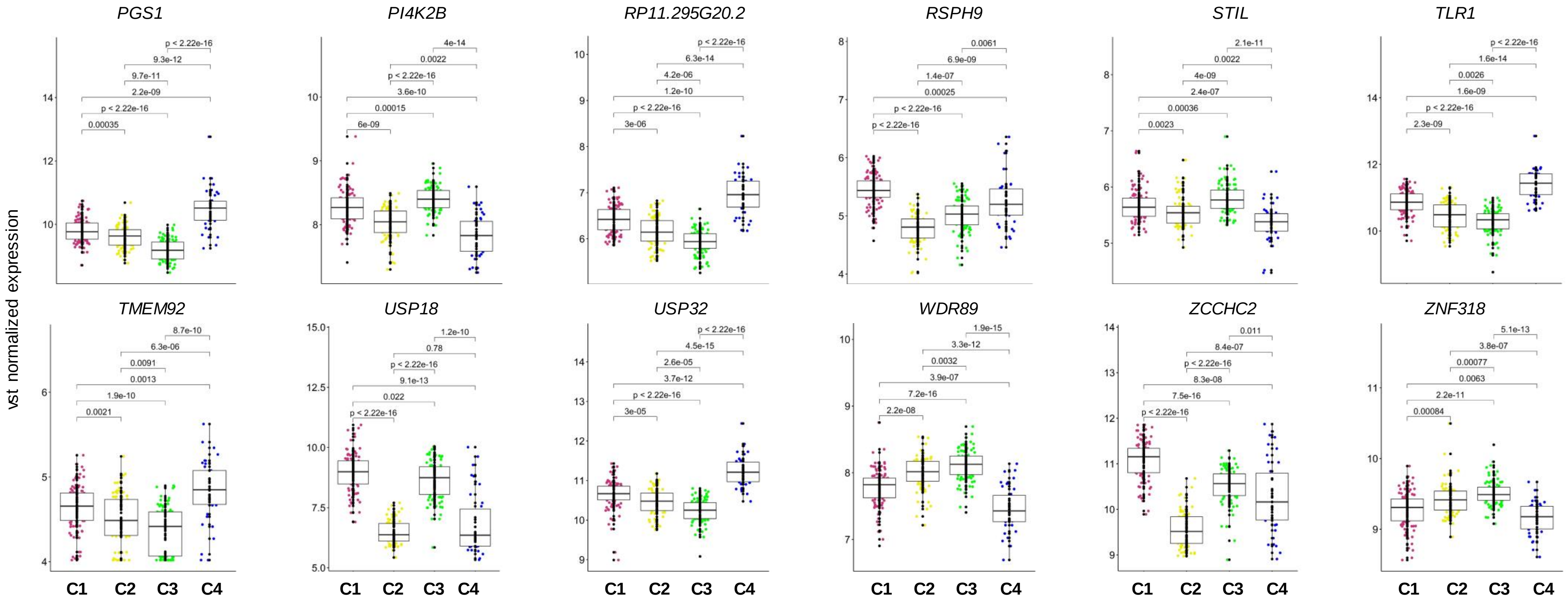

Supplementary Fig. 11: Boxplot representation of the transcripts used in the xgboost-tree for cluster prediction. Boxplots show the variance stabilizing transformation (vst) normalized expression for the 10 transcripts identified for C4 prediction (**a**) and for the 31 transcripts identified for C1, C2 and C3 prediction with *MMP25* in common for the 2 predictions (**b**) in 304 patients (C1: 101, C2: 77, C3: 88 and C4: 38). Statistical significance is determined by two-tailed pairwise Wilcoxon-rank sum test. Plots show median with error bars indicating ± interquartile range (IQR).

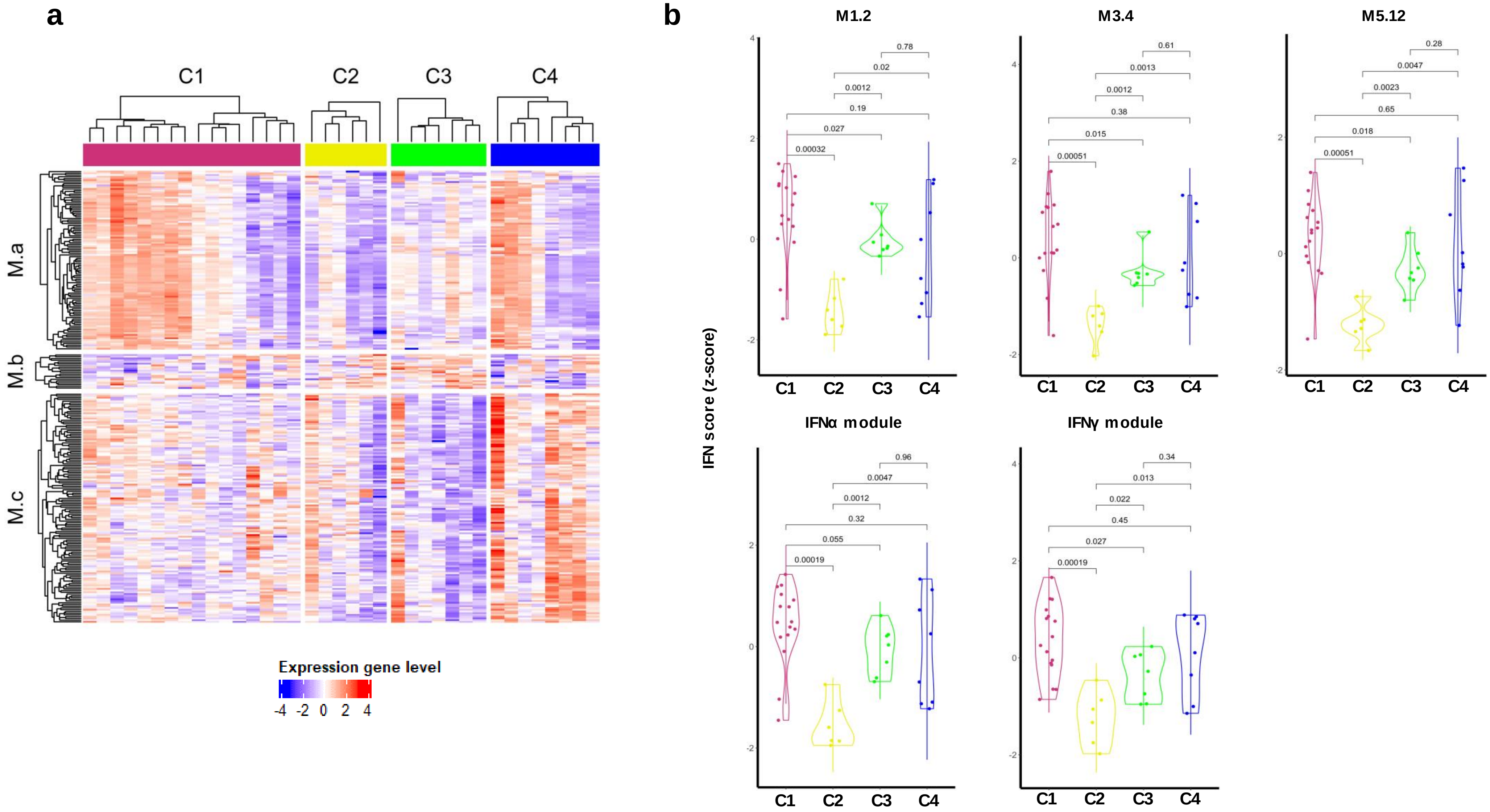

Supplementary Fig. 12: Validation of the meta model for clustering prediction in the 37 pSS patients from the inception cohort. (**a**) Heatmap performed on 37 pSS patients showing the distribution of gene transcripts across the 4 clusters (see Fig. 1A for comparison). Red represents gene over-expression and blue represents gene under-expression. (**b**) Repartition of samples from the 4 pSS clusters (C1: 16, C2: 6, C3: 7, C4: 8) are shown according to the most characterized IFN module z-scores (see Fig. 3 for comparison). Two-tailed pairwise Wilcoxon-rank sum test results is shown. Plots show median with error bars indicating the standard deviation.

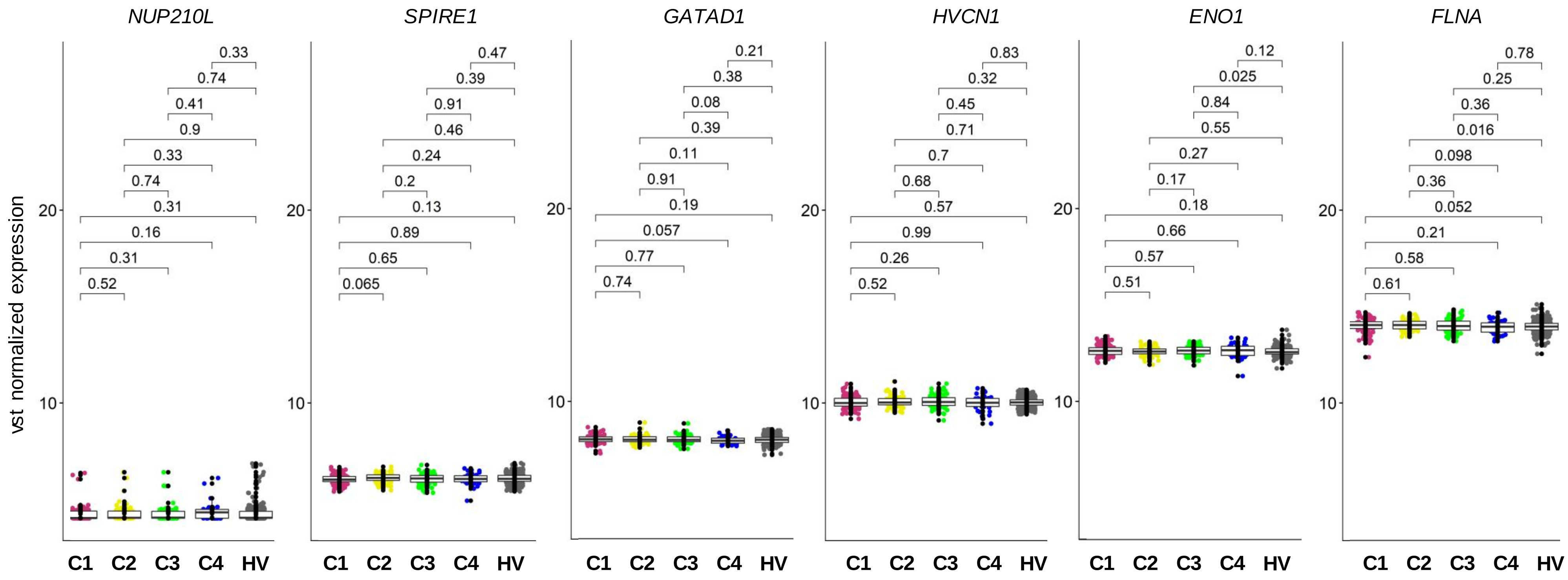

Supplementary Fig. 13: Transcripts used for interpolation function. Six genes with fold change ≤ 1.1 and false discovery rate p-value ≥ 0.05 were selected based on their constant expression across the 4 clusters (C1: 101, C2: 77, C3: 88 and C4: 38) and 330 healthy volunteers (HV). Their variance stabilizing transformation (vst) normalized expression vary between 4 and 14 [*SPIRE* (4), *NUP210L* (6), *GATAD1* (8), *HVCN1* (10), *ENO* (12) and *FLNA* (14)]. Statistical significance was determined by two-tailed pairwise Wilcoxon-rank sum test. Plots show median with error bars indicating ± interquartile range.

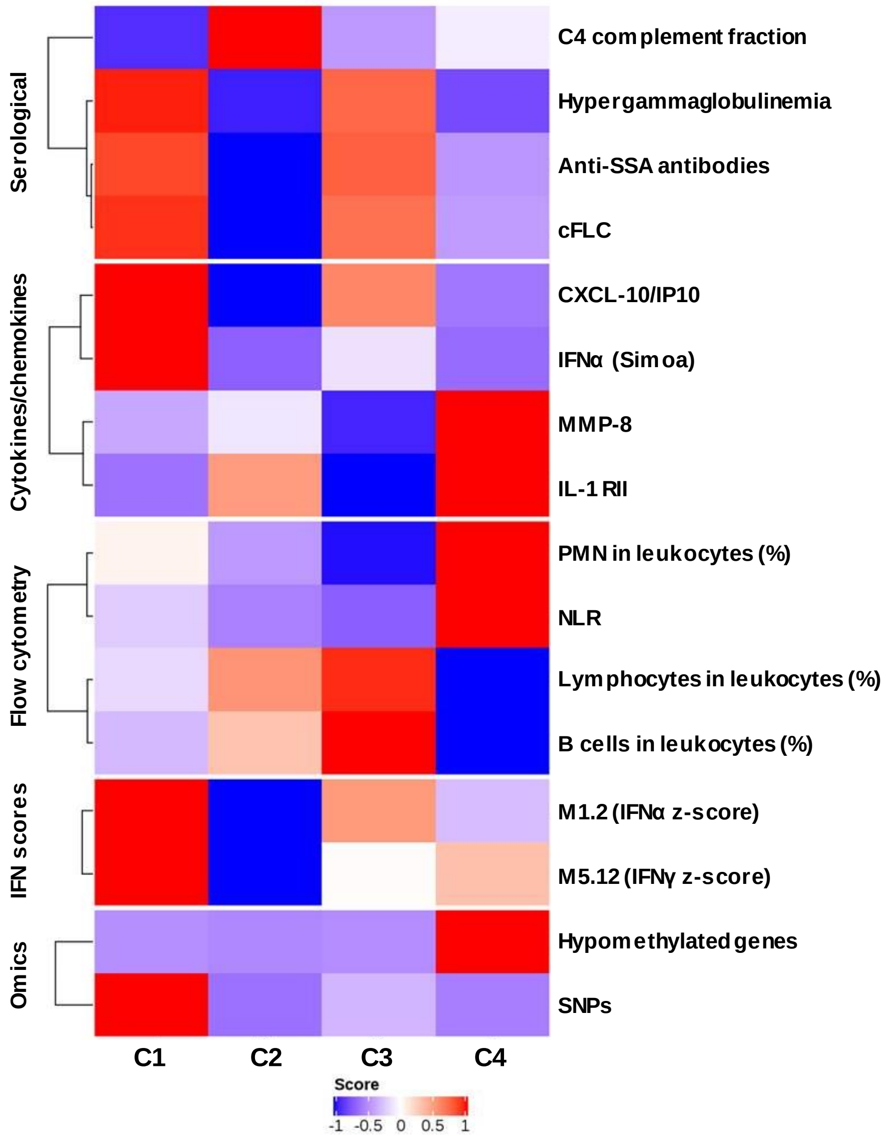

Supplementary Fig. 14: Heatmap performed on pSS patients showing the main biomarkers (with available information) able to clearly differentiate the 4 pSS clusters. cFLC: circulating free light chains, NLR: Neutrophil-to-lymphocyte ratio, SNPs: single nucleotide polymorphisms. Each biomarker has been scaled ($\frac{x-mean(x)}{sd(x)}$) to have comparable measures. Red represents biomarker over-expression and blue biomarker under-expression.

a

	FITC	PE	PC5.5	PC7	APC	APC-AF-750	PB	KO
Panel 1	CD16 (3G8)	CD15 (80H5)	CD56 (N901)	CD14 (RMO52)	CD19 (J4.119)	CD3 (UCHT1)	CD4 (13B8.2)	CD8 (B9.11)
Panel 2	CD1c (L161)	Lineage (various)	CD141 (M80)	CD11c (BU15)	CD123 (SSDCLY4107D2)	DRAQ7	HLA-DR (IMMU357)	

b

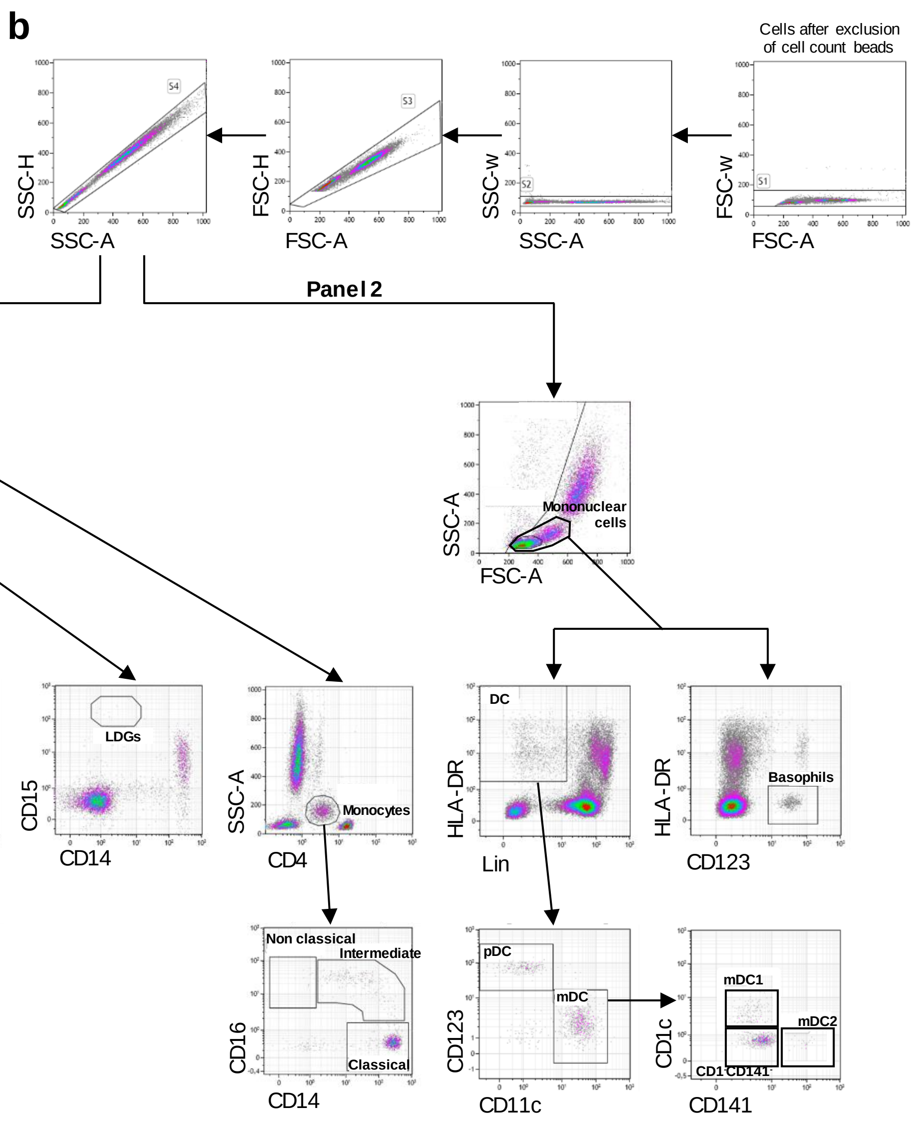

Supplementary Fig. 15: Antibody panels and gating strategies used for cell subset identifications. (**a**) Antibody panels and clones used. (**b**) Gating strategy according to the two panels of antibodies used for the immunophenotyping of PMN, Leucocytes, Mononuclear cells and Lymphocytes presented on Fig. 6 and Supplementary Fig. 7. With Panel 1, Neutrophils ($CD15^{hi}CD16^{hi}$) and Eosinophils ($CD15^{hi}CD16^{+}$) are gated from PMN. Lymphocytes are identified as B cells ($CD19^{+}CD3^{-}$), T cells ($CD19^{-}CD3^{+}$), NK cells ($CD56^{+}CD3^{-}$) and NK-like T cells ($CD56^{+}CD3^{+}$). Four T cell subsets are further gated after exclusion of NK-like T cells, the CD8+ T cells ($CD8^{+}CD4^{-}$), CD4+ T cells ($CD8^{-}CD4^{+}$), $CD4^{+}CD8^{+}$ T cells and $CD4^{-}CD8^{-}$ T cells. The $CD56^{hi}CD16^{lo}$ NK cells and $CD56^{lo}CD16^{hi}$ NK cells are identified from NK cells. LDGs ($CD14^{+}CD15^{hi}$) are gated within the Mononuclear cells. Monocytes ($CD4^{lo}$) are gated from leucocytes as classical ($CD14^{++}CD16^{-}$), intermediate ($CD14^{++}CD16^{+}$) and non-classical ($CD14^{+}CD16^{++}$) subsets. With Panel 2, DCs ($HLA\text{-}DR^{+}Lin^{-}$) and Basophils ($HLA\text{-}DR^{-}CD123^{+}$) are gated from Mononuclear cells. Within DC are identified pDC ($CD11c^{-}CD123^{+}$) and mDC ($CD11c^{+}CD123^{-}$) and three mDC subsets are further gated as $CD1^{-}CD141^{-}$ mDC, mDC1 ($CD141^{-}CD1c^{+}$) and mDC2 ($CD141^{+}CD1c^{-}$).

Algorithm 1: SjTree script

<u>Result:</u> CSV file containing clusters assignation for each patient in input file, representation of clusters distribution and linear discriminant analysis animation.

```
> Parse the user input;
if input file exist AND all mandatory columns are present then
|       > Reformat input file (select only the mandatory variables);
|       if interpolation option is NOT set to False then
|       |       > Perform interpolation on input dataset;
|       end
|       > Run cluster prediction using Algorithm 2;
|       > Create cluster distribution figure;
|       > Create LDA animation;
|       > Quit program;
else
|       > Raise warning;
|       > Quit program;
end
```

Algorithm 2: Cluster prediction

<u>Result:</u> CSV file containing clusters assignation for each patient in input file.

```
> Load dataset;
> Load model C4;
> create empty not C4 dataset;
> Run cluster prediction with model C4 on dataset;
for each patient in dataset do
|       if patient is predicted as C4 then
|       |       > Assign patient to C4 cluster;
|       else
|       |       > Add patient to not C4 dataset;
|       end
end
> Load model C2C3C3;
> Run cluster prediction with model C1C2C3 on not C4 dataset;
for each patient in not C4 dataset do
|       if patient is predicted as C1 then
|       |       > Assign patient to C1 cluster;
|       end
|       if patient is predicted as C2 then
|       |       > Assign patient to C2 cluster;
|       end
|       if patient is predicted as C3 then
|       |       > Assign patient to C3 cluster;
|       end
end

> Assemble prediction from C4 model and C1C2C3 model;
> Save assembled prediction in a csv file;
```

Supplementary Fig. 16: Description of the pseudocode for the composite model.

References

1- Chaussabel, D et al. A modular analysis framework for blood genomics studies: application to systemic lupus erythematosus. *Immunity* **29**,150-64 (2008).

2- Rinchai, D et al. BloodGen3Module: Blood transcriptional module repertoire analysis and visualization using R. *Bioinformatics* **btab121**, 1–8. (2021) https://doi.org/10.1093/bioinformatics/btab121

3- Altman, M. C. et al. A Novel Repertoire of Blood Transcriptome Modules Based on Co-expression Patterns Across Sixteen Disease and Physiological States. Preprint (2019) at https://doi.org/10.1101/525709 (2019)

4- Le Lann, L. et al. Standardization procedure for flow cytometry data harmonization in prospective multicenter studies. *Sci Rep*. **10**, 11567 (2020).

5- Bodewes, I. L. A. et al. Systemic interferon type I and type II signatures in primary Sjögren's syndrome reveal differences in biological disease activity. *Rheumatology (Oxford)* **57**, 921-930 (2018).

6- Kirou, K. A. et al. Coordinate overexpression of interferon-alpha-induced genes in systemic lupus erythematosus. *Arthritis Rheum*. **50**, 3958-67 (2004).

Supplementary note: STROBE Statement—Checklist of items that should be included in reports of ***cross-sectional studies***

	Item No	Recommendation	Relevant text from manuscript
**Title and abstract**	1	(*a*) Indicate the study's design with a commonly used term in the title or the abstract	Done in the abstract
		(*b*) Provide in the abstract an informative and balanced summary of what was done and what was found	Here, we report, in a cross-sectional cohort, a molecular classification scheme for Sjögren's syndrome patients based on the multi-omic profiling of whole blood samples from a European cohort of over 300 patients, and a similar number of age and gender-matched healthy volunteers
**Introduction**			
Background/rationale	2	Explain the scientific background and rationale for the investigation being reported	Primary SS is one of the few prototypic diseases to link autoimmunity, cancer development and infections, offering unique insights in many areas of basic science and clinical medicine. However, the pathogenesis of the disease remains elusive. Specifically, limited knowledge of existing pSS disease variants arguably represents the greatest obstacle to improve patients' diagnosis and identify patients' subsets in view of early stratification and personalized treatment
Objectives	3	State specific objectives, including any prespecified hypotheses	The present study was undertaken to establish a precise molecular classification of patients affected by pSS into more homogeneous clusters whatever their disease phenotypes, activity or treatment.
**Methods**			
Study design	4	Present key elements of study design early in the paper	In Introduction: We report herein on the integrated molecular profiling of 304 pSS patients compared to 330 matched healthy volunteers (HV) performed using high-throughput multi-omics data collected within the PRECISESADS IMI JU project (genetic, epigenomic, transcriptomic, combined with flow cytometric data, multiplexed cytokines, as well as classical serology and clinical data).
Setting	5	Describe the setting, locations, and relevant dates, including periods of recruitment, exposure, follow-up, and data collection	Recruitment was performed between December 2014 and October 2017 involving 19 institutions in 9 countries (Austria, Belgium, France, Germany, Hungary, Italy, Portugal, Spain and Switzerland).
Participants	6	(*a*) Give the eligibility criteria, and the sources and methods	Choice of the patient analysis set is detailed in Supplementary Fig. 1a.

		of selection of participants	
Variables	7	Clearly define all outcomes, exposures, predictors, potential confounders, and effect modifiers. Give diagnostic criteria, if applicable	Diagnosis of pSS was made according to the 2002 American-European Consensus Group classification criteria, with at least the presence of anti-SSA and/or a positive focus on a minor salivary gland biopsy.
Data sources/ measurement	8*	For each variable of interest, give sources of data and details of methods of assessment (measurement). Describe comparability of assessment methods if there is more than one group	For each variable of interest, sources of data and details of methods of assessment are given in Methods section.
Bias	9	Describe any efforts to address potential sources of bias	After quality control on transcriptomics RNAseq data (described below), verification of the ARC/EULAR classification criteria (focus score ≥ 1 foci/mm² and anti-SSA/Ro antibody positivity) and match of the HV to the patients based on age and gender, our final study cohort comprises 304 patients with pSS and 330 HV. This selection is detailed in Supplementary Fig. 1. Among the 304 pSS, 227 (75%) were used for the discovery step and 77 (25%) were kept for validation (Table 1).
Study size	10	Explain how the study size was arrived at	This selection is detailed in Supplementary Fig. 1.
Quantitative variables	11	Explain how quantitative variables were handled in the analyses. If applicable, describe which groupings were chosen and why	Everything is detailed in the corresponding section of Methods.
Statistical methods	12	(*a*) Describe all statistical methods, including those used to control for confounding	Everything is detailed in the corresponding section of Methods.
		(*b*) Describe any methods used to examine subgroups and interactions	Everything is detailed in the corresponding section of Methods.
		(*c*) Explain how missing data were addressed	Everything is detailed in the corresponding section of Methods.
		(*d*) If applicable, describe analytical	Not applicable

		methods taking account of sampling strategy	
		(e) Describe any sensitivity analyses	Described in Methods.
**Results**			
Participants	13*	(a) Report numbers of individuals at each stage of study—eg numbers potentially eligible, examined for eligibility, confirmed eligible, included in the study, completing follow-up, and analysed	Numbers are reported in every tables, figures and supplementary data.
		(b) Give reasons for non-participation at each stage	Numbers of missing data are reported in every tables, figures and supplementary data.
		(c) Consider use of a flow diagram	Given in Supplementary Fig. 1
Descriptive data	14*	(a) Give characteristics of study participants (eg demographic, clinical, social) and information on exposures and potential confounders	Available in Table 1 and 2.
		(b) Indicate number of participants with missing data for each variable of interest	Numbers of missing data are reported in every tables, figures and supplementary data.
Outcome data	15*	Report numbers of outcome events or summary measures	
Main results	16	(*a*) Give unadjusted estimates and, if applicable, confounder-adjusted estimates and their precision (eg, 95% confidence interval). Make clear which confounders were adjusted for and why they were included	Done when applicable
		(*b*) Report category boundaries when	Done when applicable

		continuous variables were categorized	
		(*c*) If relevant, consider translating estimates of relative risk into absolute risk for a meaningful time period	Not relevant
Other analyses	17	Report other analyses done—eg analyses of subgroups and interactions, and sensitivity analyses	Done when applicable
**Discussion**			
Key results	18	Summarise key results with reference to study objectives	Because the main current challenge in clinical trials of new therapies for pSS is the selection of the appropriate patients, we propose here a combination of molecular parameters allowing patient classification by endotypes (Supplementary Fig. 14).
Limitations	19	Discuss limitations of the study, taking into account sources of potential bias or imprecision. Discuss both direction and magnitude of any potential bias	Areas requiring further investigation have been identified. First, although our identified cluster gene signatures are strong enough to overcome the disequilibrium in blood cell counts and are not associated with disease duration, except for C2, RNA-Seq analysis is oblivious to sample cell-type composition. Further analyses are on-going, using deconvolution approaches. Second, as hypotheses were derived from a cross-sectional study and a small inception cohort, findings need to be confirmed in longitudinal cohorts to clarify whether patients will stay longitudinally in their initial cluster whatever the disease activity level and the treatments received, or whether treatments decrease disease activity by modifying the extent and scope of gene signalling dysregulations.
Interpretation	20	Give a cautious overall interpretation of results considering objectives, limitations, multiplicity of analyses, results from similar studies, and other relevant evidence	Done in the discussion.
Generalisability	21	Discuss the generalisability (external validity) of the study results	Done in the discussion.
**Other information**			
Funding	22	Give the source of funding and the role of the funders for the present study and, if applicable, for the original study on	The research leading to these results has received support from the Innovative Medicines Initiative Joint Undertaking under the Grant Agreement Number 115565 (PRECISESADS project), resources of which are composed of financial contribution from the European Union's Seventh Framework Program (FP7/2007–2013) and EFPIA companies' in-kind contribution. LBAI was supported by the Agence Nationale de la Recherche under the

	which the present article is based	"Investissement d'Avenir" program with the Reference ANR-11-LABX-0016-001 (Labex IGO) and the Région Bretagne.

*Give information separately for exposed and unexposed groups.
