# Supplementary material for: A new molecular classification to drive precision treatment strategies in primary Sjögren’s syndrome
Source: Nat Commun. 2021 Jun 10;12:3523. doi: 10.1038/s41467-021-23472-7 (PMC8192578; doi:10.1038/s41467-021-23472-7)
Supplement: Supplementary file 3 — Description of Additional Supplementary Files [file 41467_2021_23472_MOESM3_ESM.pdf]

Description of Additional Supplementary Files

File Name: Supplementary Data 1
Description: Significant differentially expressed genes (DEG) performed for 304 pSS patients from Clusters (C1: 101, C2: 77, C3: 88, C4: 38) vs 330 healthy volunteers (HV). A limma model was performed on vst transformation gene expression dataset. Resulting p-values were adjusted for multiple hypothesis testing (Benjamin-Hochberg) and filtered to retain differentially expressed genes (DEG) with a False Discovery Rate (FDR) adjusted p-value ≤ 0.05 and a |Fold-Change (FC)| ≥ 1.5

File Name: Supplementary Data 2
Description: Top 20 canonical pathways of each DEG signature in the various pSS clusters. Ingenuity pathway analysis was applied (NA : not applicable). Statistical significance was determined by Fisher's exact test.

File Name: Supplementary Data 3
Description: Significant SNPs in C2. GWAS analysis was performed using Plink, an open-source whole genome association analysis toolset, using a logistical regression on 88 pSS from C2 cluster and 330 HV. Common SNP between C3 and C1 is highlighted in red.

File Name: Supplementary Data 4
Description: IFNα association with ESSDAI domains. Distribution of ESSDAI domains (Glandular, Articular, Cutaneous, Respiratory, Renal, Muscular, Peripheral nervous, Central nervous system, Hematological, Biological) was perfomed on 78 pSS patients according to IFNα status. A patient was IFN high (hi-IFNα) if his concentration of IFNα was above the limit of quantification (49.20 fg/mL) and IFN low (lo-IFNα) if below the limit of quantification. Statisctal significance was determined by a Fisher-exact test of independance. The statistics presented are number and percent.

File Name: Supplementary Data 5
Description: Diease-specific characteristics by clusters. ESSDAI, ESSPRI and pSS main clinical characteristics were only assessed in expert centers (Barcelona, Brest, Cordoba, Geneva, Hannover, Leuven, Milano, Porto and Szeged) in a subset of 193 (C1: 70, C2: 52, C3: 44, C4: 27) of the 304 pSS studied patients. Statistical significance was determined by Kruskal-Wallis test for numeric variables and Fisher's exact test for categorical variables. The statistics presented are median and interquartile range (IQR) for continous values and numbers and percentages for categorical values.

File Name: Supplementary Data 6
Description: Anti-SSA antibody association with ESSDAI and ESSPRI in C2. Distribution of ESSDAI and ESSPRI performed on 52 and 43 pSS patients respectively following the anti-SSA antibody status. ESSPRI is the mean of three components (Pain, Fatigue and Dryness). Statistical significance was determined by a two-sided Wilcoxon rank-sum test. The statistics presented are median and interquartile range (IQR).

File Name: Supplementary Data 7
Description: Patient clinical characteristics by cluster. Distribution of clinical characteritics performed on 304 pSS patients (C1: 101, C2: 77, C3: 88, C4: 38). Statistical significance was determined by Kruskal-Wallis test for numerical variables and by Fisher's exact test for categorical variables. The statistics presented are median and interquartile range (IQR) for continous values and numbers and percentages for categorical values.

File Name: Supplementary Data 8
Description: Descriptive statistics of autoantibody expression in the different clusters. Statistical significance was determined by Kruskal-Wallis test for numeric variables and Pearson's Chi-squared test for categorical variables. The statistics presented are median and interquartile range (IQR) for continous values and numbers and percentages for categorical values.

File Name: Supplementary Data 9
Description: Probability table to belong to one of the 4 clusters assigned by the composite model for the 227 patients of the discovery set. The two columns Non-C4 and C4 prediction probabilities display the output of the first model, discriminating between C4 and all the other clusters. The columns C1, C2 and C3 prediction probabilities display the output of the second model, discriminating between cluster C1, C2 and C3. The second model is only triggered if the first model does not identify the patient in C4. Finally, the Predicted cluster column displays the final output of the composite model, assigning a cluster to each patient and the expected column corresponds to the cluster assignation in the discovery set. Discrepancies between predicted and expected cluster are highlighted in red. (N/A: not applicable).

File Name: Supplementary Data 10
Description: Probability table to belong to one of the 4 clusters assigned by the composite model for the 37 patients of the inception cohort. The two columns Non-C4 and C4 prediction probabilities display the output of the first model, discriminating between C4 and all the other clusters. The columns C1, C2 and C3 prediction probabilities display the output of the second model, discriminating between cluster C1, C2 and C3. The second model is only triggered if the first model does not identify the patient in C4. Finally, the Prediction column displays the final output of the composite model, assigning a cluster to each patient. (N/A: not applicable).

File Name: Supplementary Data 11
Description: Numbers of differentially methylated positions (DMPs) and genes obtained with 4 different thresholds of Δbeta. Whole blood methylation analysis was performed for 226 pSS patients (C1: 81, C2: 57, C3: 62 and C4: 26) and 175 healthy volunteers (HV) doing pairwise comparison between each cluster and HV applying a Benjamini Hochberg FDR < 0.1 and 4 different thesholds of Δbeta (0.05, 0.075,0.1 and 0.15).

File Name: Supplementary Data 12
Description: Mediane distribution of blood cell subsets in frequency (0-100%). Cell subset distribution was assessed by flow cytometry for 283 patients (C1: 96, C2: 71, C3: 80 and C4: 36) and 309 HV. Statistical significance was determined by Kruskal-Wallis test. The statistics presented are median and IQR.

File Name: Supplementary Data 13
Description: Median distribution of blood cell subsets in absolute numbers. Cell subset distribution was assessed by flow cytometry for 283 patients (C1: 96, C2: 71, C3: 80 and C4: 36) and 309 HV. Statistical significance was determined by Kruskal-Wallis test. The statistics presented are median and interquartile range (IQR).

File Name: Supplementary Data 14
Description: Descriptive statistics of cytokine expression in the different clusters. Cytokine expressions were mesured on serum samples for 283 patients (C1: 67, C2: 48, C3: 61 and C4: 16) and 170 HV. CXCL13/BLC, FAS Ligand, GDF15, CXCL10/IP-10, MCP-2, MCP-4, MIP-1, MMP-8, CCL17/TARC, IL-1 RII,

TNF-RI and IL1-RA were measured using the Luminex system. Soluble MMP-2, CRP, TNFα, IL-6, BAFF and TGFβ were measured using ELISA assay.Statistical significance was determined by Kruskal-Wallis test. The statistics presented are median and interquartile range (IQR).
