# Supplementary material for: A new molecular classification to drive precision treatment strategies in primary Sjögren’s syndrome
Source: Nat Commun. 2021 Jun 10;12:3523. doi: 10.1038/s41467-021-23472-7 (PMC8192578; doi:10.1038/s41467-021-23472-7)
Supplement: Supplementary file 18 — Reporting Summary [file 41467_2021_23472_MOESM18_ESM.pdf]

nature research

Corresponding author(s): Pers JO

Last updated by author(s): Apr 28, 2021

Reporting Summary

Nature Research wishes to improve the reproducibility of the work that we publish. This form provides structure for consistency and transparency in reporting. For further information on Nature Research policies, see our Editorial Policies and the Editorial Policy Checklist.

Statistics

For all statistical analyses, confirm that the following items are present in the figure legend, table legend, main text, or Methods section.

n/a	Confirmed	
☐	☒	The exact sample size (*n*) for each experimental group/condition, given as a discrete number and unit of measurement
☐	☒	A statement on whether measurements were taken from distinct samples or whether the same sample was measured repeatedly
☐	☒	The statistical test(s) used AND whether they are one- or two-sided<br>*Only common tests should be described solely by name; describe more complex techniques in the Methods section.*
☐	☒	A description of all covariates tested
☐	☒	A description of any assumptions or corrections, such as tests of normality and adjustment for multiple comparisons
☐	☒	A full description of the statistical parameters including central tendency (e.g. means) or other basic estimates (e.g. regression coefficient) AND variation (e.g. standard deviation) or associated estimates of uncertainty (e.g. confidence intervals)
☐	☒	For null hypothesis testing, the test statistic (e.g. *F*, *t*, *r*) with confidence intervals, effect sizes, degrees of freedom and *P* value noted<br>*Give P values as exact values whenever suitable.*
☒	☐	For Bayesian analysis, information on the choice of priors and Markov chain Monte Carlo settings
☒	☐	For hierarchical and complex designs, identification of the appropriate level for tests and full reporting of outcomes
☐	☒	Estimates of effect sizes (e.g. Cohen's *d*, Pearson's *r*), indicating how they were calculated

Our web collection on statistics for biologists contains articles on many of the points above.

Software and code

Policy information about availability of computer code

Data collection	No software used for the data collection
Data analysis	Except when indicated, data analyses were carried out using either an assortment of R system software (http://www.R-project.org, V4.0.1) packages including those of Bioconductor or original R code. R packages are indicated when appropriate.<br>In order to analyze RNAseq data, first, bcl2fastq2 Conversion Software v2.20 was used to demultiplex sequencing data and convert BCL files. Quality control was obtained with FastQC tools v0.11.18 and adapters were removed with Cutadapt v1.18.<br>Transcriptome alignment was done with STAR v2.5.2b on GENCODE v19 annotation (hg19) and read counts were obtained with RSEM v1.2.31.<br>For normalizations and batch correction, read counts were normalized by the variance stabilizing transformation vst function from DESeq2 v1.30.0 R package. To reduce the effect of the RIN, a correction was applied using the ComBat function from sva v3.38.0 R package, after categorization of RIN values into 7 classes: (7.5,8], (8.5,9], (9.5,10], (8,8.5], (7,7.5], (9,9.5], (6.5,7].<br>To determine the number of clusters of patients, a consensus clustering between three methods was performed: (i) Agglomerative Hierarchical Clustering (hclust function from stats v4.0.2 R package) with Pearson correlation as a similarity measure and the Ward's linkage method, (ii) K-means clustering (kmeans function from stats R package) with 4 groups and (iii) Gaussian mixture clustering (mclust function from mclust v5.4.6 R package).<br>The 257 top discriminating genes were defined with randomForest function from randomForest v4.6-14 R package.<br>Heatmap were obtained with ComplexHeatmap v2.6.2 R package.<br>To identify genes differentially expressed between pSS subgroups and HV, we performed a linear model (lmFit function from limma v3.46.0 R package) on vst transformation gene expression dataset. Resulting p-values were adjusted for multiple hypothesis testing and filtered to retain DE genes with a False Discovery Rate (FDR) adjusted p-value ≤ 0.05 and a |Fold-Change (FC)| ≥ 1.5.<br>Enrichment analysis was performed with BloodGen3Module v0.99.36 R package.<br>Canonical pathway analysis was performed with Ingenuity Pathway Analysis (IPA, Release Date: 2020-06-01).<br>Genome-wide association studies (GWAS) were performed using PLINK v1.945. The basic association for a cluster trait locus, based on comparing allele frequency between patients from each cluster vs HV, was conducted with this toolset thanks to computational resources

from the Roscoff Bioinformatics platform ABiMS. SNP annotations and Manhattan plot were obtained using the web-based tool SNPsnap from the Broad Institute and qqman v0.1.8 R packages respectively.
For methylation data, sample QC and functional normalization were completed using minfi v3.3 R-package. Briefly, during QC steps, subjects were removed based on outliers for methylated vs unmethylated signals, deviation from mean values at control probes, and high proportion of undetected probes (using minfi default parameters). DNA methylation probes that overlapped with SNPs (dbSNPs v147), located in sexual chromosomes or considered cross-reactive were removed. To identify differentially methylated positions (DMPs), the champ.DMP function of ChAMP v2.18.3 R package was implemented doing pairwise comparison between each cluster and HV.
For network viewing, we tested gene lists onto the STRING v11.0 Network of Known and Predicted Protein-Protein Interactions.
Reactome analysis of the functional pathways were performed with Pathway browser version 3.7 et Reactome database release 73.
For flow cytometry, the strategy developed to avoid any redundancy in the different cell subsets and to increase the accuracy of the phenotypes has been automated by AltraBio (Lyon, France).
The differential cytokine concentration between subgroups vs HV performed using a one-way ANOVA followed by post-hoc Tukey's test (function ghlt from multicomp v1.4-13 R package).
For the Composite model, we used the Boruta algorithm (v0.3) on all dataset to extract features that significantly contributed to predict the patient's cluster. Machine learning approaches were carried out using python programs (v3.8.5) based on the following modules: numpy (v1.18.5), scikit learn (v0.23.2) and xgboost (v1.2.0). The composite model is integrated into an analysis tool available on the laboratory's github repository at the following address: https://lbai-infolab.github.io/SjTree/
Tables with statistical analysis for flow cytometry, clinical data, autoantibodies and cytokines were obtained with stats v4.0.2 R package and gtsummary v1.3.6 R package.
Boxplot were generated with ggpubr v0.4.0 R package.
The Venn diagram were obtained with VennDiagramm v1.6.20 R package.
The plotLD function from Oncofunco v0.0.0.9000 R package was used to generate the linkage disequilibrium plot.

For manuscripts utilizing custom algorithms or software that are central to the research but not yet described in published literature, software must be made available to editors and reviewers. We strongly encourage code deposition in a community repository (e.g. GitHub). See the Nature Research guidelines for submitting code & software for further information.

Data

Policy information about availability of data

All manuscripts must include a data availability statement. This statement should provide the following information, where applicable:

- Accession codes, unique identifiers, or web links for publicly available datasets
- A list of figures that have associated raw data
- A description of any restrictions on data availability

Data that support the findings of this study have been deposited inELIXIR Data is hosted by ELIXIR Luxembourg(https://elixir-luxembourg.org/).
Data is available upon request with the identifierd access procedure is described on the ELIXIR data landing page (https://r3lab.uni.lu/frozen/th9v-xt85). The permalink is doi:10.17881/th9v-xt85.
The PRECISESADS Consortium committed to secure patient data access through the ELIXIR platform. This commitment was formerly given by written to all patients at the end of the project and to the involved Ethical Committees. The future use of the Project database was framed according to the scope of the patient information and consent forms, where use of patient data is limited to scientific research in autoimmune diseases. ELIXIR reviews applicants requests and prepares Data Access Committee's decisions on access to Data, communicates such decisions to the Data Providers, who have 10 days to exercise their right to veto; otherwise access is granted to the User.

Field-specific reporting

Please select the one below that is the best fit for your research. If you are not sure, read the appropriate sections before making your selection.

☒ Life sciences ☐ Behavioural & social sciences ☐ Ecological, evolutionary & environmental sciences

For a reference copy of the document with all sections, see nature.com/documents/nr-reporting-summary-flat.pdf

Life sciences study design

All studies must disclose on these points even when the disclosure is negative.

Sample size

Sample size has been calculated for the main PRECISESADS project as followed (see Study Protocol below):
The study of distribution of expression value between 2 groups of patients allowed to generate hypotheses for the following parameters :

Type I error probability = 0.05
Type II error probability = 0.20 (i.e. Power 1 – = 0.80)
Two-tailed tests
Allocation ratio = 9 (i.e. the cluster accounts for 10% of the total population)
Effect sizes: For t-tests on expression data: Cohen's d = 0.2 (i.e. difference of the means divided by standard deviation = 20%)

With these parameters, sample size estimations give: n1 = 218 and n2 = 1966. Total N = 2184.

Where n1 is the sample size of the cluster, n2 the sample size of the remaining population, and N = n1 + n2 the total sample size.

	On average, a total sample size of approximately 2000 patients (400 per disease) will allow identifying clusters of minimum 200 patients.<br><br>In this study population comprised 382 primary Sjögren's syndrome (pSS) patients and 341 Healthy Volunteers enrolled in the main PRECISESADS cross-sectional study.
Data exclusions	Following complete quality control and diagnosis validation (in accordance with the 2016 ACR-EULAR classification criteria of the disease, each patient had to present either anti-SSA/Ro antibody positivity or focal lymphocytic sialadenitis with a focus score of ≥1 foci/mm2), 78 patients were removed. 11 HV, with autoantibodies, were also removed.
Replication	RNA-Sequencing data obtained from whole blood were not replicated.<br>However, to perform the clustering of the remaining 304 samples, samples were divided into a discovery set and an independent validation set, representing 75% and 25% of samples, respectively. The discovery set allowed to cluster patients in four groups, as confirmed in the validation set.<br>No replication of other experiments was performed.
Randomization	Randomization was not relevant since this is an observational study. Consequently, we have filled the STROBE statement enclosed in the manuscript as a supplementary note.<br>For this study, 330 healthy volunteers from 617 initially have been matched on pSS population using the age and the gender.
Blinding	Blinding was not feasible since this is an observational study.

Reporting for specific materials, systems and methods

We require information from authors about some types of materials, experimental systems and methods used in many studies. Here, indicate whether each material, system or method listed is relevant to your study. If you are not sure if a list item applies to your research, read the appropriate section before selecting a response.

Materials & experimental systems

n/a	Involved in the study
☐	☒ Antibodies
☒	☐ Eukaryotic cell lines
☒	☐ Palaeontology and archaeology
☒	☐ Animals and other organisms
☐	☒ Human research participants
☐	☒ Clinical data
☒	☐ Dual use research of concern

Methods

n/a	Involved in the study
☒	☐ ChIP-seq
☐	☒ Flow cytometry
☒	☐ MRI-based neuroimaging

Antibodies

Antibodies used	Two panels of antibodies were used for flow cytometry:<br><br>Panel 1: Duraclone ref B38675 (Beckman Coulter, Bangalore, India)<br>CD16-FITC (IgG1 clone 3G8) / CD15-PE (IgM clone 80H5) / CD56-PC5.5 (IgG1 clone N901) / CD14-PC7 (IgG2a clone RMO52) / CD19-APC (IgG1 clone J3.119) / CD3-APCA750 (IgG1 clone UCHT1) / CD4-PBE (IgG1 clone13B8.2) / CD8-KrO (IgG1 clone B9.11)<br>Lot number used in the study: 13BDC228-2, 13BDC228-3 and 13BDC228-6<br><br>Panel 2 : Duraclone ref B38685 (Beckman Coulter, Bangalore, India)<br>CD1c-FITC (IgG1 clone L161) / Lineage-PE (IgG1 CD3 clone UCHT1, IgG1 CD19 clone J3.119, IgG2a CD14 clone RMO2, IgG2a CD20 B9E9 (HCR20), IgG1 CD56 clone N901) / CD141-PC5.5 (clone M80) / CD11c-PC7 (clone BU15) / CD123-APC (clone SSDCLY107D2) / DRAQ7-APCA750 / HLA DR-PBE (clone IMMU357)<br>Lot number used in the study: 13BDC229-2, 13BDC229-3 and 13BDC229-6
Validation	The Duraclone panels include optimized antibody combinations for the identification and characterization of cells of the human immune system. Dry, unitized antibody panels, powered by DURA Innovations technology (https://www.beckman.com/resources/technologies/dura-innovations), include backbone markers for cell identification, opens slots for drop-in markers, and save time on designing immunophenotyping assays (https://www.beckman.com/reagents/coulter-flow-cytometry/antibodies-and-kits/duraclone-panels)<br><br>Testing was performed on normal whole blood and/or cultured cell lines to confirm reactivity and specificity of each antibody within the combinations.<br><br>Relevant citations:<br>Hedley BD, Keeney M, Popma J, Chin-Yee I. Novel lymphocyte screening tube using dried monoclonal antibody reagents. Cytometry B Clin Cytom. 2015 Nov-Dec;88(6):361-70. doi: 10.1002/cyto.b.21251. Epub 2015 Jul 17. PMID: 25944189.<br>Bouriche L, Bernot D, Nivaggioni V, Arnoux I, Loosveld M. Detection of Minimal Residual Disease in B Cell Acute Lymphoblastic Leukemia Using an Eight-Color Tube with Dried Antibody Reagents. Cytometry B Clin Cytom. 2019 Mar;96(2):158-163. doi: 10.1002/cyto.b.21766. Epub 2019 Jan 30. PMID: 30698327.

Beckman Coulter warrant that the product will perform in accordance with the performance criteria in the product data sheet used and stored as set forth in the product data sheet.

Human research participants

Policy information about studies involving human research participants

Population characteristics	Patients with primary Sjögren's Syndrome (pSS) and healthy controls (HV) were obtained from the European multi-center cross-sectionnal study of the PRECISESADS IMI consortium. Eligible patients were diagnosed within less than a year since pSS diagnosis. W analyzed 304 pSS patients and 330 matched HV for age (58.401 ± 13.448 vs 53.294 ± 10.998) and gender (92.76% of females vs 91.52 in HV group), recruited in 18 institutions from 9 European countries and enrolled in the cross-sectional cohort of the PRECISESADS IMI project.
Recruitment	Recruitment was performed between December 2014 and October 2017 involving 19 institutions in 9 countries (Austria, Belgium, France, Germany, Hungary, Italy, Portugal, Spain and Switzerland). The Ethical Review Boards of the 18 participating institutions approved the protocol of the cross-sectional study. The inception study used to validate the composite model was also obtained from the PRECISESADS Consortium. Inception patients were recruited by 10 institutions in Spain, Belgium, France, Italy, Germany and Switzerland.<br>No bias of selection has been identified.
Ethics oversight	The two studies (cross-sectional and inception) adhered to the standards set by International Conference on Harmonization and Good Clinical Practice (ICH-GCP), and to the ethical principles that have their origin in the Declaration of Helsinki (2013). Each patient signed an informed consent prior to study inclusion. The Ethical Review Boards of the 19 participating institutions approved the protocol of the cross-sectional study. Moreover, the protocol of the inception study was approved by the ethical committees of the 10 participating institutions. These 10 sites were also participating to the cross-sectional study, therefore these ethical committees reviewed both protocols. The ethical committees involved were: Comitato Etico Milano, Italy; Comité de Protection des Personnes Ouest VI Brest, France; Comité d`Èthique Hospitalo-Facultaire de Louvain, Belgium; Comissao de ética para a Saude –CES do CHP Porto, Portugal; Comité Ética de Investigación Clínica del Hospital Clínic de Barcelona, Spain; Commissie Medische Ethiek UZ KU Leuven /Onderzoek, Belgium; Geschaftsstelle Ethikkommission,Cologne, Germany; Ethikkommission Hannover, Germany; Ethik Kommission. Borschkegasse, Vienna, Austria; Comité de Ética e la Investigación de Centro de Granada, Spain; Commission Cantonale d`éthique de la recherche Hopitaux universitaires de Genève, Switzerland; Csongrad Megyei Kormanyhivatal, Szeged, Hungary; Ethikkommission, Berlin, Germany; Andalusian Public Health System Biobank, Granada, Spain. The protection of the confidentiality of records that could identify the included subjects is ensured as definedby the EU Directive 2001/20/EC and the applicable national and international requirements relating to data protection in each participating country. The cross-sectional and inception studies are registered in ClinicalTrials.com with respectively number NCT02890121 and number NCT02890134.

Note that full information on the approval of the study protocol must also be provided in the manuscript.

Clinical data

Policy information about clinical studies

All manuscripts should comply with the ICMJEguidelines for publication of clinical research and a completedCONSORT checklist must be included with all submissions.

Clinical trial registration	The cross-sectional and inception studies are registered in ClinicalTrials.com with respectively number NCT02890121 and number NCT02890134.
Study protocol	Protocol Code: PRECISESADS CS<br>GA-115565<br>Molecular Reclassification to Find Clinically Useful Biomarkers for Systemic Autoimmune Diseases<br><br>Date: 03/11/2014<br><br>Fundacion Progreso y Salud<br>Avda. Américo Vespucio 5, Bloque 2, 2ª Planta<br>Parque Científico y Tecnológico Cartuja 41092 - Sevilla<br><br>The information in this document is confidential and it is property of the PRECISESADS Consortium, operating under the INNOVATIVE MEDICINES INITIATIVE JOINT UNDERTAKING (IMI-JU). Therefore, the information should not be disclosed, published, made public or otherwise transferred to a third party, in any form without written permission from the PRECISESADS Consortium. However, this document may be disclosed to researchers and potential researchers, health authorities and relevant national ethics committees under the condition that they respect the confidential nature of this document.<br><br>Table of Contents<br><br>AMENDMENTS AND UPDATES<br>ABBREVIATIONS OF TERMS USED<br>RESPONSIBLE PARTIES<br>Centers for post-recruitment sample handling and laboratory analysis<br>Other responsible parties

PROTOCOL SUMMARY
1. Study Title.
2. Protocol Code.
3. Coordinating Investigator and contact info.
4. Recruiting Clinical Sites .
5. Objectives.
6. Design.
7. Schedule of Events
Clinical assessments for the different populations of the study are detailed below:
8. Diseases at study.
9. Subjects
10. Study Procedures
11. Study Duration.
12. Financial sources.
MAIN OBJECTIVE AND SPECIFIC OBJECTIVES.
1. Main Objective
2. Specific Objectives
BACKGROUND.
METHODS.
1. Enrollment of Patients
2. Subjects and Study size.
3. Data sources and measurement.
4. Variables.
5. Data transformation.
6. Statistical Methods.
7. Quality control.
8. Bias.
ETHICAL ISSUES /Protection of participating subjects
The Protocol and informed consent forms will be approved by independent Ethics Committee
1. Participating subjects benefits-risks assessment.
2. Informed consent forms
3. Confidentiality statement.
4. Potential Interference with current practice and prescriptions of medicine.
5. Ethical Monitoring and final study reports.
RESULTS DISSEMINATION PLAN.
FINANCIAL SOURCES.
BIBLIOGRAPHY
ANNEXES

ABBREVIATIONS OF TERMS USED

Anti-CCP Anti-citrullinated peptide antibodies
ChIP-SEQ Chromatin immunoprecipitation sequencing
GCP Good Clinical Practice
GWAS Genome-wide association study
IRB/IEC Institutional Review Board/ Independent EthicsCommittee
LC Liquid chromatography
LIMS Laboratory Information Management System
MS Mass spectrometry
MCTD Mixed Connective Tissue Disease
NMR Nuclear magnetic resonance
NGS Next generation sequencing
PAPS Primary antiphospholipid syndrome
RA Rheumatoid arthritis
RNA-SEQ Transcriptome sequencing with NGS methods
SAD Systemic Autoimmune Disease
SLE Systemic lupus erythematosus
SSc Scleroderma orsystemic sclerosis
SjS Sjögren’s syndrome
WPL Work Package Leader
WPcL Work Package co-Leader

Institution Abbreviations
ALTHIA Advanced Laboratories of Translational Health
BAYER Bayer AG Holding Company
CHP Centro Hospitalar do Porto.
CING The Cyprus Foundation for Muscular Dystrophy Research
CSIC Consejo Superior de Investigaciones Científicas

DRFZ Deutsches Rheuma-Forschungszentrum Berlin
LILLY Eli-Lilly and Company
FPS Fundación Pública Andaluza Progreso y Salud
SARD Genzyme Corporation, a Sanofi company
HPORTO Centro Hospitalar do Porto
HUG Hospitaux Universitaires de Géneve
IDIBAPS Institut d'Investigacions Biomèdiques August Pi i Sunyer
IDIBELL Institut d'investigacio Biomedica de Bellvitge
IRCCS Fondazione IRCCS Ca Granda Ospedale Maggiore Policlinico
IRIS Institut de Recherches Internationales : Servier
KI Karolinska Institutet
KU LEUVEN Katholieke Universiteit Leuven
MHH Medizinische Hochschule Hannover
MUW Medizinische Universität Wien
QBIO QuartzBIO
SAS Servicio Andaluz de Salud
SCS Servicio Cantabro de Salud
UBO Université de la Bretagne Occidentale
UCB UCB Biopharma SPRL
UCL Université Catholique de Louvain
UGR Universidad de Granada
UKK Klinikum der Universitaet zu Koeln
UNIGE Université de Genève
UNIMI Università degli studi di Milano
USZ University of Szeged

RESPONSIBLE PARTIES

Centers for post-recruitment sample handling and laboratory analysis:
FPS
CSIC
IRCCS
CHP
SAS - Cordoba
IDIBELL
KI
KU LEUVEN
MHH
UBO
UNIGE
UNIMI
DRFZ

Other responsible parties:

Gene expression analysis of exosomes: Althia, FPS
Analysis – gene expression : Bayer, FPS
Bioinformatics & Statistics: Quartz BIO, IDIBELL, FPS, Bayer, CSIC, UBO
Clinical Data Management : IRIS

PROTOCOL SUMMARY

1. Study Title.
Molecular Reclassification to Find Clinically Useful Biomarkers for Systemic Autoimmune Diseases: Cross Sectional Cohort (PRECISESADS)
2. Protocol Code.
PRECISESADS GA-115565_CS
3. Coordinating Investigator and contact info.
Prof. Marta E. Alarcón Riquelme,
Fundación Pública Andaluza Progreso y Salud
GENYO: Center for Genomics and Oncological Research
Avda de la Ilustración 114
PTS 18016, Granada,
Spain
Tel: +34 958 715 500 ext 113
Mobile phone: +34 671595280
Email: Marta.alarcon@genyo.es

4. Recruiting Clinical Sites (19).
1-Centro Hospitalar do Porto, Largo Prof. Abel Salazar 4099-001 PORTO (Portugal)
2-Fondazione IRCCS Ca Granda Ospedale Maggiore Policlinico via Francesco Sforza n.28 20122 Milano (Italy)
3-Hospital Clinic I Provicia- Institut d'Investigacions Biomèdiques August Pi i Sunyer Calle Villarroel 170 08036 Barcelona (Spain)
4-Hospital Universitario San Cecilio Servicio Andaluz de Salud Avda. del Dr. Oloriz nº16 18012 Granada (Spain)
5-Hospital Universitario Reina Sofía Andaluz de Salud Avda. Menéndez Pidal, s/n 14004 Córdoba (Spain)
6-Hospital Universitario Marqués de Valdecilla, Servicio Cántabro de Salud Avd. Cardenal Herrera Oria s/n, 39011 Santander, (Spain)
7-UNIMI, Istituto Ortopedico Getano Pini, Piazza A. Ferrari 1, 20122 Milano (Italy)
8-University of Szeged, H-6720 Szeged, Dugonics square 13 (Hungary)
9-Medical University of Vienna Spitalgasse 23, 1090 Wien (Austria)
10-Hospital Regional de Málaga Servicio Andaluz de Salud Avda. Carlos Haya s/n 29010 Málaga (Spain)
11-Hospitaux Universitaires de Géneve Rue Gabrielle-Perret-Gentil 4, 1205 Genève (Switzerland)
12-Centre Hospitalier Universitaire de Brest Hospital de la Cavale Blanche Boulevard Tanguy Prigent CP : 29609 Brest CEDEX, (France)
13-UZ Leuven - KU Leuven, Department of Rheumatology Herestraat 49, 3000 Leuven (Belgium)
14-Deutsches Rheuma-Forschungszentrum Berlin Charitestraße 1, 10117 Berlin (Germany)
15-Medizinische Hochschule Hannover Carl-Neuberg-Str. 1 30625 Hannover (Germany)
16-Hospital Virgen de las Nieves Granada Avenida de las Fuerzas Armadas, 2, 18014, Granada (Spain)
17-Université catholique de Louvain – Cliniques Universitaires Saint-Luc Avenue Hippocrate 10, 1200 Brussels (Belgium)
18-University of Cologne, Dept. of Dermatology, Kerpener Str. 62, 50937, Cologne (Germany)
19-Andalusian Public Health System Biobank, Granada (Spain).

5. Objectives.
The main objective of the PRECISESADS project is to reclassify the individuals affected by SADs into molecular clusters instead of clinical entities through the determination of molecular profiles using several "-omics" techniques.
The specific objectives of this cross sectional study and sub-study are:
a) To identify a systemic taxonomy for patients with SADs by producing the following data in individuals with SADs and controls: genetic, epigenomic, transcriptomic, flow cytometric (from peripheral blood mononuclear and polymorphonuclear cells (PBMCs)), metabolomics and proteomic in plasma and urine, exosome analysis, classical serology (antibodies and autoantibodies), and clinical data.
b) To better characterize individual SADs at the omics level.
c) To perform clustering analyses to determine the groups of individuals who, differentially from other groups, share specific molecular features (precision medicine).
d) To identify gene expression, methylation profiles through deconvolution methods comparing a mixture of cells with subpopulations determined by flow cytometry with separated cells, cytokine profiles and plasma metabolomics using Mass Spectrometry, in a substudy of 288 individuals.
The clustering process will be data-driven with the aim to find the most homogenous and differentiated clusters of diseases that clearly separate individuals on the basis of, serological, genetic, epigenomic, cellular (cell proportions), metabolomic, proteomic (cytokines, autoantibodies) and transcriptome characteristics and differentiate them from controls and other patient clusters.

6. Design.
This is a European multi center, non randomized, cross sectional clinical study aiming at collecting clinical and biological data on patients and healthy controls with systemic auto immune diseases.

7. Schedule of Events
Clinical assessments for the different populations of the study are detailed below:

n=400 SLE patients
n=400 RA Patients
n=400 SSC patients
n=400 Sjs patients
n=400 Paps/MCTD patients
n=666 Healthy controls (matched)

Informed consent for all
Inclusion/exclusion criteria for all
Demography for all
Clinical diagnosis for all
Clinical history for all
Disease activity for all
Current treatment for all
Clinical data (lab & Imaging data when available) for all
Clinical examination for all
Blood sample for all
Urine samplefor all
Separated Cell Substudy blood sampling *** optional that depends on specific informed consent

8. Diseases at study.

Connective tissue diseases:
o Systemic lupus erythematosus
o Rheumatoid arthritis
o Scleroderma or systemic sclerosis
o Sjögren’s syndrome
o Primary antiphospholipid syndrome
o Mixed Connective Tissue Disease
o Patients with undifferentiated disease

9. Subjects
A total of 2000 patients and 666 controls will be included in the study, adjusted to the following distribution:
o A total of 400 patients diagnosed with systemic lupus erythematosus (SLE)
o A total of 400 patients diagnosed with rheumatoid arthritis (RA)
o A total of 400 patients diagnosed of scleroderma or systemic sclerosis (SSc)
o A total of 400 patients diagnosed of Sjögren’s syndrome (SjS)
o A total of 400 patients diagnosed of primary antiphospholipid syndrome (PAPS) or Mixed Connective Tissue Disease (MCTD) or with undifferentiated disease
For the sub study on “Separated Cell Populations”
• A subset of 288 subjects will be included in this substudy: 48 patients of each disease category as described above and 48 controls.
• All patients will be recruited from 18 sites in Europe (Austria, Belgium, France, Germany, Italy, Portugal, Spain, Hungary and Switzerland).
10. Study Procedures
For 2666 individuals (2000 cases, and 666 controls):
a) Fresh whole blood for flow cytometry analyses (2ml);
b) Blood for processing to obtain plasma and DNA (20ml);
c) Blood for RNA (5ml);
d) Blood for serum (17ml);
e) Blood for lupus anticoagulant (2.7ml)
f) Urine (100 ml).
representing, a total of approximately 50 ml of blood taken in one single extraction .

Sub-study on “Separated Cell Populations”
A selected set of 288 individuals (240 patients, 48/disease and 48 controls) will be studied in more detail by allowing cell separation in selected centres which will be done to:
- determine differences in gene expression and epigenetic marks in the separated cells as compared to total blood
-optimize deconvolution methods which determine gene expression and methylation profiles in a blood mixture where the cell types are defined in parallel using flow cytometry.
For these measurements, the following additional samples will be collected:
-Fresh blood for cell separation (an extra 80-100 ml)
Thus, this sub group of individuals will have a total of 130-150 ml of blood taken, as possible, with only one extraction.

11. Study Duration.
Recruitment from September 2014 /August 2017
Total study Duration from September 2014 to January 2019

12. Financial sources.
The research leading to these results has received support from the Innovative Medicines Initiative Joint Undertaking under grant agreement n°115565, resources of which are composed of financial contribution from the European Union's Seventh Framework Programme (FP7/2007-2013) and EFPIA companies’ in kind contribution.
WORK FLOW AND MILESTONES.

Month-Year Duties Responsible
February 2014-May 2014 Preparation of protocols and instructions FPS, Lilly, IRCCS, IRIS, IDIBAPS
June 2014 – September 2014 Ethics Committee Submissions Each center
September 2014-August 2017 Recruitment period

Collection of samples and
laboratory processing of samples Principal investigators of all clinical sites
SAS (Granada and Córdoba sites), UBO, IRCCS, UNIGE, UK LEUVEN, UCL, DRFZ, and IDIBAPS, participate in the recruitment of patients for the substudy as well.

Laboratory sample processing by: The Andalusian Biobank.
FPS, BAYER, DRFZ, IDIBELL, CSIC, KU LEUVEN, ALTHIA, KI, UGR, UNIMI, UBO
January 2015-May 2015 Data analysis of first sets of processed materials, and optimization of protocols according to preliminary results Coordinating investigator and sample analysis sites : FPS, IDIBELL, DRFZ, UBO, KI, CSIC, ALTHIA, UGR, UNIMI, BAYER, QBIO
January 2015-December 2017 Analysis of samples and data by research sites (bioinformatical or genetic analysis, statistical) FPS, IRIS, QBIO, IDIBELL, UBO, UGR, CSIC, ALTHIA, DRFZ, BAYER, KI, UKLEUVEN, UNIMI
January 2015-January 2019 Data analysis ongoing throughout the project and dissemination of the results FPS, UCB, CSIC, IDIBELL, UBO, DRFZ, QBIO, BAYER, IRIS, SARDS, KI, UNIMI

MAIN OBJECTIVE AND SPECIFIC OBJECTIVES.

1. Main Objective
To reclassify the individuals affected by SADs into molecular clusters, instead of clinical entities through the determination of molecular profiles using several "-omics" techniques.

2. Specific Objectives
To identify a systemic taxonomy for patients with SADs by producing the following data in individuals with SADs and controls: genetic, epigenomic, transcriptomic, flow cytometric (from peripheral blood mononuclear cells (PBMCs)), metabolomics and proteomic in plasma and urine, exosome analysis, classical serology (antibodies and autoantibodies), and clinical data
To better characterize individual SADs at the omics level.
To perform clustering analyses to determine the groups of individuals who, differentially from other groups, share specific molecular features (precision medicine)
To identify gene expression, methylation profiles through deconvolution methods comparing a mixture of cells with subpopulations determined by flow cytometry with separated cells, cytokine profiles and plasma metabolomics using Mass Spectrometry, in a substudy of 288 individuals
The clustering process will be data-driven with the aim to find the most homogenous and differentiated clusters of diseases that clearly separate individuals on the basis of, serological, genetic, epigenomic, cellular (cell proportions), metabolomic, proteomic (cytokines, autoantibodies) and transcriptome characteristics and differentiate them from controls and other patient clusters.

BACKGROUND.

Connective tissue diseases (CTD) or systemic autoimmune diseases (SADs) as they are known today are a group of chronic inflammatory conditions with autoimmune aetiology with few treatment options and difficult diagnosis. Their major common feature is the presence of unspecific autoantibodies in serum. Three diseases primarily represent the SADs: systemic lupus erythematosus (SLE), rheumatoid arthritis (RA) and systemic sclerosis (SSc) that show extensive overlap in their presentation. Several other entities and syndromes have extensive clinical overlap with these, where Mixed Connective Tissue Disease (MCTD), Sjögren's syndrome (SSj) and the primary antiphospholipid antibody syndrome (PAPS) are very relevant examples. While as separate clinical entities each of these diseases is rare, together they make up to close to 1% of the general population. In addition there are individuals who do not fulfil the clinical criteria or who do not share all the features of a given clinical entity and live for years as undifferentiated cases. These diseases are the focus of our attention.

Mortality and morbidity associated with these disorders is high, and there is a need for more adequate therapies, which either stop progression of the disease, or prevent the occurrence of flares and organ damage. However, the development of such therapies is presently limited by insufficient knowledge of the complex mechanisms involved in these diseases, and, more importantly, of the mechanisms that are activated in individual cases. Thus, the development of a new molecular taxonomy of these disorders will meet this "medical need" in patients suffering from such disorders while utilising high-throughput molecular methods.

As a prototype, new promising biological treatments are being available or developed for SLE (i.e. Belimumab, Atacicept, Epratuzumab, to name some), but because of separate disease classification, their use cannot benefit other diseases where shared molecular pathophysiology is suspected on scientific grounds. Currently patients are being exposed to novel and approved agents with little chance of benefit due to the heterogeneity of molecular mechanisms resulting in the same disease class or patients are being denied access to potentially beneficial novel and approved agents due to misclassification to a different disease class despite similar aetiological mechanism. Furthermore, pharmaceutical companies confront huge problems when attempting to identify end points to determine the usefulness of drugs in clinical trials and lack biomarkers that help evaluate response to therapy.

The PRECISESADS project is a joint venture bringing together 21 academic institutions across Europe, 2 biotechnology companies, and 5 Pharmaceutical companies with the aim of performing a new classification of the following systemic autoimmune diseases: systemic lupus erythematosus, systemic sclerosis or scleroderma, Sjögren's syndrome and rheumatoid arthritis. primary antiphospholipid syndrome, mixed connective tissue disease and cases of patients that are undifferentiated because they do not fulfill criteria for any of the former are also included. Genetic, epigenetic, transcriptomic, metabolomics and serological analysis will be performed to identify, independently of their clinical diagnoses, clusters that would differentiate a given set of individuals from the others. Clinical and demographic data using a monitorized electronic case report form (eCRF) to correlate with the molecular clusters will also be collected.

In addition, the study will help to advance important pieces of work such as the genetics of systemic autoimmune diseases and the study on disease pathways, genetic analyses between genotypes and several parameters will be performed: metabolite profiles and metabolite levels, methylation profiles, gene expression quantitative traits (eQTLs), cell subpopulation numbers and proportions, cytokine levels, autoantibody specificities. The traits will be analyzed for specific diseases, across diseases and/or specific clusters, and/or combined with clinical data.

METHODS.

1. Enrollment of Patients

Inclusion criteria

For all patients

• Caucasian patients and aged 18 years or older at the time of consent
• Diagnosed according to prevailing criteria for one of the following systemic autoimmune diseases (see Annex 2)

o Rheumatoid arthritis (RA)
o Scleroderma or systemic sclerosis (SSc)
o Primary Sjögren's syndrome (SjS)
o Systemic lupus erythematosus (SLE)
o Primary antiphospholipid syndrome (PAPS)
o Mixed Connective Tissue Disease (MCTD)
o Patients with undifferentiated connective tissue disease (UCTD) for over 1 year and that do not fulfill the diagnosis of any of the above diseases.
• Signed the informed consent form

For controls

• Will be matched on the projected and expected profile of the patients in terms of gender, age and clinical center of origin

Exclusion criteria

For all patients

• Patients unable to understand the procedures related to the protocol should not be included. The study is voluntary and patients must be able to give their informed consent.
• Pregnant women
• Neonatal lupus
• Drug-induced lupus
• Patients whose condition is so serious that they cannot take part in the study
• Severe nephrotic syndrome with proteinuria ≥ 3,5 g/day
Patients with stable doses of steroids >15mg/day for the last 3 months or with IV corticosteroids in the last 3 months
• Patients under immunosuppressants for the last 3 months prior to recruitment with:
o Methotrexate ≥25mg/week
o Azathioprine ≥2.5mg/kg/day
o Cyclosporine A > 3mg/kg/day
o Mycophenolate Mofetil > 2gr/day
• Treatment with cyclophosphamide (any dose or route of administration) or Belimumab in the past 6 months
• Patients with combined therapy of two or more immunosuppressants
• Patients on depletative therapy such as Rituximab in the last year
• Patients receiving experimental therapy.
• Chronic HBV or HCV infection
• Overlap syndromes
NOTE: Definitions are provided in annex 2

For controls
• Individuals on chronic medication.
• Individuals suffering from any inflammatory, autoimmune, allergic or infectious condition, and if possible without a history of autoimmune disease, particularly thyroid disease or other diseases that may modify cellular profiles in blood.
• Pregnant women.

2. Subjects and Study size.
The complete study will recruit a total of 2000 patients and 666 controls.
The sub study will consist of a sub set of 288 individuals (240 patients, 48/disease and 48 controls).

Statistical analysis of clustering: Patients will be clustered based on their biomarker data measures. Density-Based Spatial Clustering of Applications with Noise (DBSCAN) types of methods will be adapted to account for the heterogeneity of data types (e.g. categorical data, continuous data, ratios), the biotechnological features of their acquisition (e.g. sample stability, dynamic ranges, batch effects, normalization effects), the internal inter-dependencies in data (e.g. genetics vs. gene expression, whole blood gene expression vs. cell counts), the big number of data points, and the biological annotations and prior knowledge on data.

Once a cluster is identified, the main biomarkers that differentiate that cluster from the remaining patients' population will be analyzed for association: The distribution of expression values (resp. genotype data) of these biomarkers in the cluster of interest will be compared with the distribution in the remaining population by the mean of a t-test for differences between two independent groups (resp. Fisher exact test). The following features are expected:
- Type I error probability = 0.05
- Type II error probability = 0.20 (i.e. Power 1 – = 0.80)
- Two-tailed tests
- Allocation ratio = 9 (i.e. the cluster accounts for 10% of the total population)
- Effect sizes:
o For t-tests on expression data: Cohen's d = 0.2 (i.e. difference of the means divided by standard deviation = 20%)
o For Fisher tests on genotype data: Difference of proportion of the minor allele of 10%, e.g. 35% vs. 25%
With these parameters, sample size estimations give:
- For t-tests on expression data: n1 = 218 and n2 = 1966. Total N = 2184.
- For Fisher tests on genotype data: n1 = 192 and n2 = 1728. Total N = 1920.

Where n1 is the sample size of the cluster, n2 the sample size of the remaining population, and N = n1 + n2 the total sample size.
On average, a total sample size of approximately 2000 patients will allow identifying clusters of minimum 200 patients defined by biomarkers that differentiate those clusters from the remaining population with an effect size of 0.2 for continuous expression data and 10% for genotype data, with type I error 5% and statistical power 80%.
Analysis of gene expression and other omics comparisons between patient and control data:
The substudy will concentrate on the discovery of new transcripts in separated cell populations, in particular granulocytes, T and B lymphocytes and monocytes. This part of the study will use RNA-SEQ to discover novel splice forms or microRNAs. The discovered transcripts will be studied in the larger sample with other methods (i.e. TaqMan PCR on RNA obtained from the larger sample set, depending on the discovered RNAs or splice variants, alternatively, a custom array using the complete sample).
The same is expected for the analysis of cytokines.
According to calculations done for the gene expression study and discovery of differential transcripts, the power curve displayed below (which is also valid for differential analysis of any analyte, e.g. cytokines), that will be done in the whole set of patients, allows the following assumptions: assuming a false discovery rate p=0.05, a standard deviation of 0.7, and the proportion of differentially expressed transcripts = 0.05 and using 25 subjects from each group there is ~80% power to detect a fold change (FC) of 1.75 and 98% power to detect a FC=2.0.
While we do have a problem with heterogeneity of the samples because these originate from different sites, the complete gene expression, methylation, and other analyses will be done on a single platform, minimizing heterogeneity

3. Data sources and measurement.
The following data will be produced from individuals with SADs and controls: genetic, epigenomic, transcriptomic, flow cytometric data (from PBMCs and granulocytes), plasma and urine metabolomics, serum cytokine analyses, and serology (autoantibodies).

A sample collection kit prepared by the Andalusian biobank will be provided to each of the clinical groups with all the necessary tubes for collection and processing of the samples. Tubes will be pre-labeled ("OMIC number", numbering information described below).

Tubes will be for the collection of the following samples:

a) Total blood for flow cytometry (2ml) for measurement of cell proportions with all panels: will be done on site at each clinical site with 8 or 10-color flow cytometers (all sites except MUW, SCS, USZ and SAS Málaga which will transfer the blood sample to the closest equipped site).
b) Blood for processing to obtain DNA and plasma (20 ml); the tube will processed to obtain an aliquot plasma and the cell pellet will be frozen and sent to the Andalusian biobank for the extraction of DNA. Plasma will be used for NMR metabolite measurements (this analysis requires only a few microliters).
c) Preserved blood to obtain good quality total RNA (5 ml); these tubes will be frozen and sent to the Andalusian biobank
d) Blood for processing to obtain serum (about 17 ml or two tubes of 8.5ml each); this tube will be centrifuged, serum obtained and transferred to smaller tubes aliquoted and frozen and sent to the Andalusian Biobank for storage
e) Blood with citrate for measuring lupus anticoagulant (2.7ml); this tube will be centrifuged, plasma obtained and transferred to smaller tubes and frozen to be sent to the Andalusian Biobank for storage.
e) Urine (about 100ml): Will be processed according to instructions, aliquoted and sent to the Andalusian biobank.

From the substudy individuals:

-Extra tubes of fresh blood for cell separation (about 80-100 ml); these tubes will be used in the cell separation facility of the recruitment center of selected clinical sites with capabilities to do so.

The substudy will be performed only in 7 of the recruiting clinical centers and in no more than 240 patients and 48 controls. These selected individuals will have a total of 150 ml of blood taken if possible in a single extraction (one vein), most probably in a single donation.

The following are the measurements to be performed at the analysis sites in samples obtained from all patients:

a) Gene expression and gene methylation in total blood. Gene expression will be done using commercial gene expression microarrays in total blood from all samples using the RNA Paxgene tube. Methylation analysis will be done using the methylome 450k array using the DNA obtained from total blood. MicroRNA gene expression arrays using total blood.
b) Flow cytometry analysis to determine cell proportions in the total blood mixture in all individuals. 2 optimized panels of antibodies will be used to determine cell subpopulations (including very minor cell populations). For the flow cytometry, optimization for all measurements is taking place between UBO and FPS. The company Beckman Coulter will provide lyofilized antibodies that will be sent to each group doing the flow cytometry analysis and beads to calibrate the various platforms at each site. For these analyses, 50ul of blood is enough for each panel of antibodies. Analysis data detailing flow cytometry-gating information and fluorescence intensities for each antibody/color will be transferred to UBO for the integrated analysis.
c) Genotyping will be done using a whole genome array.
d) Metabolite determination in plasma and urine using Nuclear Magnetic Resonance (NMR).
e) Exosome isolation from plasma and urine: set up of the methodology for isolating exosomes in these bodily fluids for gene expression analysis.
f) Cytokine profile determination across diseases and differences with controls.
g) To determine routine autoantibodies in serum: a set of serum autoantibodies will be determined in a European validated laboratory. Also, they will perform detection of antibodies against small lipid moieties (i.e.anti-phosphorylcholine), lupus anticoagulant and complement proteins in plasma.

The analyses to be performed in samples from the patients (n=240) and controls (n=48) of the substudy will be the following:

a) Blood will be obtained for isolation of neutrophils, and separation of peripheral blood mononuclear cells into CD3+ T cells, CD19+ B cells and CD14+ monocytes.
b) Flow cytometry profiles using 8 panels of antibodies to determine all possible cell subpopulations.
c) Gene expression in separated cell populations using RNA-SEQ and gene expression microarrays for discovery of novel splice variants, isoforms of genes and transcripts that may be tissue/cell specific using next generation sequencing methodologies. From the separated cells, RNA-SEQ will be used to reveal new microRNAs, lncRNAs and mRNA not found in the literature or the databases.
d) Gene methylation in separated cell populations using the methylome 450k array.
e) Histone marks in separated cell populations. Epigenomic analyses including chromatin-IP for histone 3 methylation at lysine 4 (H3K4m3, mark for active transcription) and histone 3, lysine 27 trimethylation (mark for transcriptional repression), H3K27me3.
f) Determination of approximately 100 cytokines using Luminex assays in serum.
g) Metabolite determination and quantification in plasma and urine using MS/LC and correspondence with profiles using NMR.

Detailed instructions for the collection and processing of the samples will be provided by FPS and the Andalusian biobank in a separate laboratory manual. Once a number of samples have been collected and frozen at -80°C, these will be batched and sent frozen by courier to the Andalusian biobank.

Once received, the Andalusian Biobank will prepare the derivatives from the blood (DNA, RNA), and store plasma, urine and serum in adequate facilities with minimal documentation (diagnosis, gender, country and year of birth) provided by the recruitment center. The Biobank will then send the needed materials to the various analysis sites for processing.

The following sites will be doing the analyses listed for the cross sectional study and sub-study:

IDIBELL: Epigenomic analyses, methylome arrays.
CSIC: Genotyping
FPS: RNA-SEQ, expression microarrays, NMR for metabolomics for all samples, flow cytometry and cell separation for the samples coming from Granada centers, urinary and plasma exosome gene expression analysis.
UGR: Mass-Spectometry for metabolome in plasma and urine of substudy samples.
KI: small-lipid moiety antibodies in serum
UNIMI: cytokines in serum by LUMINEX method.
UBO: flow cytometry and cell separation for local samples and integrated analyses of flow cytometry data for all sites and determination of autoantibodies.
BAYER: Will perform expression microarrays in part of the samples.
ALTHIA: Gene expression analysis of exosomes

The following sites will be performing cell separation and 8-panel flow cytometry:

IRCCS, SAS (Hospital Reina Sofía, Córdoba), UBO, FPS, IDIBELL, UNIGE, UK LEUVEN, UCL, DRFZ

The following sites will be performing 2-panel flow cytometry:

IRCCS, SAS (Córdoba, Hospital Reina Sofía), FPS, IDIBELL, UNIGE, DRFZ, CHP, MHH, UNIMI, UK LEUVEN, UCL.

IDIBAPS, UKK, MUW, USZ and SAS (Málaga) will be recruiting patients but samples for flow cytometry will be transferred to the closest sites.
IRIS: Will monitor the clinical data through the eCRF collection

Each center will process the samples, perform primary analyses of the data and then transfer the data for integration and cluster analysis to QuartzBIO as it has been established in the internal Data Governance Plan. Data Data will be transferred via a password protected and/or encrypted secure transfer system for data protection. Data from cytokines, autoantibodies, epigenetics, genetics, NMR, flow cytometry will be also shared via secure transfer, through an intranet site for the project protected by usernames and passwords, with exception of the clinical data (see below) so that other sites can use the data to perform various analyses. All samples will only be labeled with the OMICS number.

Controls will be obtained:
For the general study: Those controls coming from Andalucía will be obtained through the Andalusian biobank.
For other centers, these will be obtained through active recruitment at each center. All prenumbered kits and tubes will be provided by the Andalusian biobank and sent to the center in charge locally. The same gender ratios and mean age (+/- 5 years) will be followed as for the patients.
For controls in the main study, 50 ml of blood will be obtained and the amount required for the flow cytometry will be sent to the local analysis associated center in charge. Thereafter, other samples will be treated exactly as the patient samples and sent to the Andalusian biobank as instructed.

For the controls of the substudy, the same procedure will take place, except that the 80-100ml for the cell separation will be sent also to the local associated center for processing.

4. Variables.
4.1 Genetic measurements: carriage of risk or protective alleles, characterization
4.2 Epigenetic measurements: differentially methylated sites as compared to controls or between diseases, or between clusters. Histone marks in cell populations

4.3 Transcriptomic measurements: differentially expressed transcripts as compared to controls or between diseases or between

clusters.

4.4 Serological measurements: differentially expressed cytokines as compared to controls, or between diseases or between clusters

4.4.1 Routine serology: battery of autoantibodies by routine methods to be done in only one validated center.
4.4.2 Other serology: antibodies to small lipid moieties in serum, differences between diseases or clusters

4.5 Clinical Data

Clinical data will be collected based on clinical examination as well as lab and imaging data when available from the patients' file (see below). De-identified clinical data will be entered in an eCRF system that will be monitored for compliance including date of informed consent signature.
Inclusion into the eCRF system, as well as any study procedures will not proceed until an ethical committee approval has been obtained and a signed informed consent form is available. The e-CRF will be produced by Institut de Recherches Internationales Servier (I.R.I.S.) in compliance with the study specifications.
The investigator or a designated person from his/her team will be trained for the use of the e-CRF and will perform the data entry at each clinical site. De-identified data will be transmitted via the Internet and stored in a secured database.
In order to ensure confidentiality and security of the data, usernames and passwords will be used to restrict system access to authorised personnel only, whether resident within the clinical sites, I.R.I.S. or third parties.
After the last visit of the participant, the investigator or co-investigator must attest the authenticity of the data collected in the e-CRF by entering his/her user name and password.
I.R.I.S. is responsible for data processing including data validation performed according to a specification data management plan describing the checks to be carried out. As a result of the data validation processes, some data may require clarifications and eventual changes. An electronic data clarification form will be sent to the investigator when this occurs.
All corrections of data on the e-CRF will be made by the investigator or by the designated person from his/her team using electronic data clarifications. All data modifications will be recorded using the audit trail feature of the software used (including date, reason for change and identification of the person who made the change).
When data validation is achieved, a review of the data will be performed. When the database has been declared to be complete and accurate, it will be locked and made available for analysis at QuartzBIO.
In order to determine if the various SADs can be re-classified independently of their clinical diagnoses or clinical criteria, clinical data across diseases will be obtained as follows:
- Demographics: gender, year of birth
- Clinical diagnosis according to the clinical site, age at the time of the first symptoms of the disease
- Clinical data related to each relevant organ class and based on clinical examination such as constitutional signs and symptoms, mucocutaneous and joint manifestations, pulmonary, gastrointestinal or cardiovascular involvement.
- Imaging results will be used when available through the patients' file. Laboratory abnormalities will be collected when available in the patients' file, but information will be also obtained in the study (e.g. autoantibodies).
- Disease activity
- Treatment of the patients including start dates?
For controls the year of birth, gender, status (control) and center or collection will be included in the eCRF by the biobank or center from where the sample is originating. Controls obtained in the Andalusian biobank will be also included in the eCRF together with the OMICs number that will be installed in the biobank.

5. Data transformation.
The data obtained at each of the laboratory analyzing centers will be then sent as processed data to QuartzBIO where it will be transformed informatically into data that can be used for the clustering analyses. Processing will depend on the type of system to be used for each laboratory analysis. The laboratories will only have available the sample code called "OMICS number" (see below) and all data will be generated using only this number.
The biomarker data management will be operated in the framework of an ISO9001-based quality management system and in compliance with established standards ensuring patient's personal data privacy and confidentiality.

Overall the process will be as follows:
- A patient (or control) number ("patient OMIC number") will be generated by the biobank LIMS system (Andalusian biobank) and the kits will be pre-labeled and sent to the clinical sites recruiting the patients.
- The OMIC number has the following structure: xxyyzzzz uuu, where
- xx is the Granada Biobank number (32).
- yy is the year in which the kit was created by the Biobank (e.g. 14 for 2014).
- zzzz is the random number correlated with the specific donation from each patient/control.

After the Biobank receives the samples, the above number is registered in the Biobank LIMS, three digits (uuu) will automatically follow according to the type of samples derived from the particular donation starting with 001 (e.g. urine or blood sample). Because of the structure of the code, including the year, this number is never repeated and its link with the donation number (xxyyzzzz) guarantees traceability.
The Biobank will also receive a worksheet where the OMIC number, the year of birth, gender and the diagnosis will be included, together with comments on the sample extraction and the confirmation that the informed consent has been signed by the patient with the patient choice relative to sample conservation at the end of the study.

- The eCRF will generate a patient (or control) number ("eCRF patient number") when the clinical data is to be entered by the physician team or by the Andalusian biobank team for the controls recruited there. The numbers of individuals reached by each center will be monitored through the eCRF.
- The sites enrolling a patient will obtain the patient informed consent with regards to study participation and sample processing by

the biobank. Then clinical data that will be introduced in the eCRF including informed consent date and patient choice with regard to sample conservation at study end.
- The physician will enter the OMIC number into the eCRF system for later correlation with the clinical data at the moment of integration of the data.
- After informed consent is obtained and documented, samples will be taken in accordance to the instructions provided with the kits and tubes.
- All samples except those for flow cytometry and/or cell separation will be returned to the Andalusian biobank together with a form that will include in addition to the “patient OMIC number” other information: center, gender, year of birth and diagnosis.
- Samples for flow cytometry and cell separation will be immediately sent to the local site for processing.
- Once the biobank has processed the samples received, the blood derivatives and the processed urine will be sent to the analysis sites that will analyze the samples. These analysis sites will generate the data and process the data, known as OMICS data. Transportation of the samples to the biobank and from the biobank will follow the WHO international guide, and special care will be taken so that samples are considered as potentially infectious using P650 packaging for dangerous goods (see lab manual).
- The OMICS data generated (the results) by the analysis laboratories will be sent to Quartz bio showing only their “patient OMIC number” and will be shared between analysis sites through an internal project server (intranet).
- Clinical data completed at the eCRF will be sent to Quartz bio when the analysis requires it, with their “OMIC number” as entered in the eCRF, to compatibilize with the OMIC data received by the analysis sites.

The data integration and management process will be performed using the TransMART software platform, optimized for OMICS data management and integration with clinical data. Only processed data (e.g. called genotypes, metabolite concentration, flow cytometry absolute counts) will be managed in this repository. The project will benefit from TransMART integration and access control capabilities when limited clinical data will need to be mapped onto clusters.

The initial setup of the data governance process will define the Standard Operating Procedure (SOP) to manage OMICS data. Only analysts trained to this SOP will have access to data.

Sharing of samples may be possible with collaborating groups in the United States and groups that perform research in SADs and that formally request samples for study, once the PRECISESADS study is completed. The informed consent contains this information .

6. Statistical Methods.
The study deals with exploratory clustering analyses. One significant part of the work is to benchmark existing clustering methods on the data structure to determine the most relevant approach. It is therefore impossible to define a priori what exact statistical methods will be used. However, the roadmap is well-defined:
1. Establish cluster metrics to evaluate cluster homogeneity and robustness, identify confounding effects, and compare clusters;
2. Test clustering algorithms (e.g. density, connectivity, and centroid models, hard and soft clustering, with and without overlapping clusters) based on these metrics on simulated datasets to evaluate how they manage heterogeneous data types, how they resist to background noise, what are their performances and possible optimizations, and what is the impact of the scaling and weighting parameters;
3. Develop customized visualization and reporting tools. The main issues that will be investigated are
a. High-dimensionality (how to cluster with many more features than observations? How to select/reduce features?);
b. Heterogeneity (How to manage continuous and categorical data at the same time? How to deal with various background noise types?);
c. Correlated structure (How to take into account the chronological and spatial structure of biological data and their underlying regulation processes?
d. How to draw strong local correlations from a global pattern of expected weak correlation)

Biomarkers or combinations of biomarkers will be modeled to explain the disease phenotype (disease vs. control, or across the different types of systemic autoimmune diseases), adjusting by available relevant covariates (classically gender, age, region, etc). Other statistical approaches (e.g. eQTL analyses, epistasis, pathway analyses) and finding of new loci with quantitative traits (cytokines, metabolites, autoantibody levels), will be tested for more specific purposes, but the data to be used will be from the complete sample. For genetic data, at this point, only mapping and filtering of sequencing data will be done to compare with public databases. This comparison is to identify novel variants across the samples. The same will be done for the identification of novel transcripts (splice forms) or microRNAs and therefore, no statistical methods are used.
Relevance of transcripts, methylation profiles, metabolite profiles, etc will be analyzed using two-sided t-tests.

In the substudy, the statistical methods used for deconvolution analyses will be mainly based on generalized linear models. Deconvolution analyses will be tested against the use of gene expression in separated cells.

7. Quality control.
To minimize potential bias, coordinated protocols for sample management will be distributed among all partners.

8. Bias.
The principal bias detected is that molecular profiling with state of the art techniques requires the protocols for isolation, fractionation, and storage of samples to be well standardized. Other potential biases are found in the treatment of the patients. Therefore we exclude patients with strong immunosuppressive therapies or cell depletative therapes (e.g. Rituximab or Belimumab) and create specific cellular profiles not corresponding to the disease process itself.

ETHICAL ISSUES /Protection of participating subjects

The Protocol and informed consent forms will be approved by each site’s independent Ethics Committee and the approval will clearly document the version and date of both the protocol and the ICF approved, as well as the principal investigator and list the study site. Copies of all EC approvals will be kept in the study master file at FPS.

1. Participating subjects benefits-risks assessment.
The participants will not have any direct benefit from their participation in this study, however the assumed risk is minimal compared to the great contribution to the development of knowledge of their disease. Minimal events due to blood sampling itself, such as hematoma or bleeding not requiring medical attention will not be reported. Serious events requiring medical attention will be reported to the approving ethics committee and the project's Ethical Advisory Board.

2. Informed consent forms
All participants included in this study must read and understand the information provided by the investigators in the clinical sites, answer the questions related to sample processing by the biobank and communication of results, and sign the informed consent form. Each patient will receive an original copy of their signed consent form.

Consent for approval to store the samples in the biobank permanently will be sought from each individual to be recruited in the informed consent form. The individual will be asked to approve or refuse the use of the samples beyond the study for public research on systemic autoimmune diseases only.

The option to have the sample destroyed if desired, at any time during the study and by the end of the study, is also included in the informed consent form.
Traceability of the sample is guaranteed through an organized LIMS system to enable destruction when requested. The Andalusian Biobank is compliant with all European regulations and norms for biobanks and Spanish Law on biobanks.

Samples will be stored, until finished at the Andalusian Health System Biobank located at the Center for Biomedical Research, located at Avda del Conocimiento s/n, Parque Tecnológico de la Salud, 18016, Granada, Spain. The Andalusian Health System biobank is under the governance of the Andalusian Health Care system and the Fundación Progreso y Salud and will therefore be responsible for all the samples. The Biobank has all the facilities for the proper storage of samples of all types (tissue biopsies, blood, serum, urine, cell lines, organs for donations) and facilities for preparing high quality DNA, RNA, etc.
* The option of storing samples for future use will be determined by each patient's consent on the ICF as well as the option to withdraw the consent for sample storage. All samples obtained from patients have been done so at no charge or payment.

After the end of the PRECISEADS project, study samples will be made available to investigators with documented experience in the study of SADs, through a system of peer-review by a Steering Board made up of PRECISESADS investigators. Prior to that, samples will only be available to investigators in the PRECISESADS project and for the objectives of this project.

3. Confidentiality statement.
The confidentiality of records that could identify subjects will be protected, respecting the privacy and confidentiality rules in accordance with the local applicable regulatory requirement(s). No personal identifiers will be introduced in the databases, and full date of birth will only be introduced completel in the eCRF if in agreement with the local regulations otherwise, only year of birth will be introduced; gender, diagnosis, and originating center of the sample as variables will be linked with the biological samples with the OMIC number described above.

4. Potential Interference with current practice and prescriptions of medicine.
This study does not require the follow-up of the participants and is not intended to interfere with the current clinical practice or subject's treatment. No new treatment will be provided and no deviations from the standard therapeutic procedures as decided by the treating physician will be introduced.

5. Ethical Monitoring and final study reports.

The PRECISESADS Consortium has established an independent Ethics Advisory Board (EAB) to monitor the ethical procedures of the project. During the course of this study, annual reports will be sent to the EAB. Reports to the local Ethics Committees will also be provided when applicable according to local regulations. At study completion, a final study report will be distributed to EAB and all approving ECs.
In case samples are sent to collaborators or third parties having a request approved by PRECISESADS, then an MTA will be established and archived at FPS. The EAB will also be informed.

FINANCIAL SOURCES.

The research leading to these results has received support from the Innovative Medicines Initiative Joint Undertaking under grant agreement n°115565, resources of which are composed of financial contribution from the European Union's Seventh Framework Programme (FP7/2007-2013) and EFPIA companies' in kind contribution.

BIBLIOGRAPHY

1. Bennett, L. et al. Interferon and granulopoiesis signatures in systemic lupus erythematosus blood. J Exp Med 197, 711-23 (2003).
2. Dall'era, M.C., Cardarelli, P.M., Preston, B.T., Witte, A. & Davis, J.C., Jr. Type I interferon correlates with serological and clinical manifestations of SLE. Ann Rheum Dis 64, 1692-7 (2005).
3. Tan, F.K. et al. Signatures of differentially regulated interferon gene expression and vasculotrophism in the peripheral blood cells of systemic sclerosis patients. Rheumatology (Oxford) 45, 694-702 (2006).
4. Li, Q.Z. et al. Interferon signature gene expression is correlated with autoantibody profiles in patients with incomplete lupus syndromes. Clinical and experimental Immunology 159, 281-91 (2010).
5. Li, Q.Z. et al. Identification of autoantibody clusters that best predict lupus disease activity using glomerular proteome arrays. J Clin Invest 115, 3428-39 (2005).

6. Depristo, M.A. et al. A framework for variation discovery and genotyping using next-generation DNA sequencing data. Nature Genetics 43, 491-8 (2011).
7. Simpson, R.J., Lim, J.W., Moritz, R.L. & Mathivanan, S. Exosomes: proteomic insights and diagnostic potential. Expert review of proteomics 6, 267-83 (2009).
8. Fang, D.Y., King, H.W., Li, J.Y. & Gleadle, J.M. Exosomes and the kidney: blaming the messenger. Nephrology 18, 1-10 (2013).
9. Delgado-Vega, A., Sanchez, E., Lofgren, S., Castillejo-Lopez, C. & Alarcon-Riquelme, M.E. Recent findings on genetics of systemic autoimmune diseases. Curr Opin Immunol 22, 698-705 (2011).
10. Yin, H. et al. Association of STAT4 and BLK, but not BANK1 or IRF5, with primary antiphospholipid syndrome. Arthritis and Rheumatism 60, 2468-71 (2009).
11. Delgado-Vega, A.M., Alarcon-Riquelme, M.E. & Kozyrev, S.V. Genetic associations in type I interferon related pathways with autoimmunity. Arthritis research & therapy 12 Suppl 1, S2 (2010).
12. Houssiau FA, et al. Azathioprine versus mycophenolate mofetil for long-term immunosuppression in lupus nephritis: results from the MAINTAIN Nephritis Trial. Ann Rheum Dis. 2010 Dec;69(12):2083-9.
13. Bertsias GK, et al; European League Against Rheumatism and European Renal Association-European Dialysis and Transplant Association. Joint European League Against Rheumatism and European Renal Association-European Dialysis and Transplant Association (EULAR/ERA-EDTA) recommendations for the management of adult and paediatric lupus nephritis. Ann Rheum Dis. 2012 Nov;71(11):1771-82.
14. Gieger, C. et al. Genetics meets metabolomics: a genome-wide association study of metabolite profiles in human serum. PLoS Genet 4, e1000282 (2008).
15. Ouyang, X., Dai, Y., Wen, J.L. & Wang, L.X. (1)H NMR-based metabolomic study of metabolic profiling for systemic lupus erythematosus. Lupus 20, 1411-20 (2011).

ANNEXES

Annex 1 Investigator assurances

According to the signed Grant Agreement (GA-115565) of the study entitled “Molecular Reclassification to Find Clinically Useful Biomarkers for Systemic Autoimmune Diseases: PRECISESADS: Cross Sectional Cohort”, the researchers will:

• Conduct the study in accordance with the relevant, current protocol.
• Personally conduct and supervise the described investigation(s).
• Ensure that all associates, colleagues, and employees assisting in the conduct of the study are properly trained and informed about their obligations in meeting the above commitments.
• Promptly report to the IRB/IEC all changes in the research activity and all unanticipated problems involving risks to human subjects or others.
• Not make any changes in the research without IRB/IEC approval, except where necessary to eliminate apparent immediate hazards to human subjects. In such a case, the EC will be informed as soon as humanly possible.
• Comply with all other requirements regarding the obligations of clinical investigators and all other pertinent requirements in tripartite harmonized ICH Guideline E6 for good clinical practice and DECLARATION OF HELSINKI Ethical Principles for Medical Research Involving Human Subjects (rev Fortaleza Brazil Oct 2013).

Annex 2: Clinical criteria

The diagnostic criteria used in the inclusion of patients will be based on the following references:
-RA: 2010 ACR/EULAR classification criteria (Ann Rheum Dis 2010;69:1580-1588)
-SLE: 1997 update of 1982 ACR criteria (Arthritis Rheum 1997;40:1725).
-SSc: ACR/EULAR 2013 classification criteria (Ann Rheum Dis. 2013;72:1747-55)
-SjS: AECG SjS classification criteria (Ann Rheum Dis 2002;61:554–8)
-MCTD: Alarcon-Segovia criteria for MCTD (J Rheumatol 1989;16:328-334)
-PAPs: International consensus statement on an update of the classification criteria for definite PAPs (J Thromb Haemost, 2006;4: 295–306)
-UCTD: patients with clinical features of SADs not fulfilling any of the above or any other SADs criteria for at least 2 years + Presence of unspecific antibodies, ANA ≥ 1:160.
Patients fulfilling 3 out of 4 SLE classification criteria and patients with Early Systemic Sclerosis (J Rheumatol. 2001, 28:1573-6) should not be classified as UCTD.

Data collection

Recruitment was performed between December 2014 and October 2017 involving 19 institutions in 9 countries (Austria,Belgium, France, Germany, Hungary, Italy, Portugal, Spain and Switzerland):
1-Centro Hospitalar do Porto, Largo Prof. Abel Salazar 4099-001 PORTO (Portugal)
2-Fondazione IRCCS Ca Granda Ospedale Maggiore Policlinico via Francesco Sforza n.28 20122 Milano (Italy)
3-Hospital Clinic I Provicia- Institut d’Investigacions Biomèdiques August Pi i Sunyer Calle Villarroel 170 08036 Barcelona (Spain)
4-Hospital Universitario San Cecilio Servicio Andaluz de Salud Avda. del Dr. Oloriz nº16 18012 Granada (Spain)
5-Hospital Universitario Reina Sofía Andaluz de Salud Avda. Menéndez Pidal, s/n 14004 Córdoba (Spain)
6-Hospital Universitario Marqués de Valdecilla, Servicio Cántabro de Salud Avd. Cardenal Herrera Oria s/n, 39011 Santander, (Spain)
7-UNIMI, Istituto Ortopedico Getano Pini, Piazza A. Ferrari 1, 20122 Milano (Italy)
8-University of Szeged, H-6720 Szeged, Dugonics square 13 (Hungary)
9-Medical University of Vienna Spitalgasse 23, 1090 Wien (Austria)
10-Hospital Regional de Málaga Servicio Andaluz de Salud Avda. Carlos Haya s/n 29010 Málaga (Spain)
11-Hospitaux Universitaires de Géneve Rue Gabrielle-Perret-Gentil 4, 1205 Genève (Switzerland)
12-Centre Hospitalier Universitaire de Brest Hospital de la Cavale Blanche Boulevard Tanguy Prigent CP : 29609 Brest CEDEX, (France)
13-UZ Leuven - KU Leuven, Department of Rheumatology Herestraat 49, 3000 Leuven (Belgium)

14-Deutsches Rheuma-Forschungszentrum Berlin Charitestraße 1, 10117 Berlin (Germany)
15-Medizinische Hochschule Hannover Carl-Neuberg-Str. 1 30625 Hannover (Germany)
16-Hospital Virgen de las Nieves Granada Avenida de las Fuerzas Armadas, 2, 18014, Granada (Spain)
17-Université catholique de Louvain – Cliniques Universitaires Saint-Luc Avenue Hippocrate 10, 1200 Brussels (Belgium)
18-University of Cologne, Dept. of Dermatology, Kerpener Str. 62, 50937, Cologne (Germany)
19-Andalusian Public Health System Biobank, Granada (Spain).

Multiomic data were collected in addition to clinical and routine biological data, including GWAS, transcriptome, methylome, flow cytometry, autoantibodies and cytokines (See Study Protocol for details).
Sample collection, processing and storage were coordinated by the Andalusian Biobank System, which is integrated into the Andalusian Health system as a way of maintaining a centralized and harmonized sample processing. To ensure harmonization and traceability of prospective biological samples, a fit for purpose centralized model for management of sample collection consumables, sample storage and distribution was developed by the project biobank. The study databases and eCRFs were designed specifically to capture data for the PRECISESADS project. The Inform eCRF was the tool used to collect data from each participating site. All data and analyses produced were stored in a project TransMart database used in collaboration with the eTRIKS IMI consortium and hosted by QuartzBio/Precision Medicine. Data has been transferred to ELIXIR and made available upon request and in agreement with the privacy rules of the European Union.

Outcomes

AAll data used for the present research were collected within the frame of observational studies. The principal objective of the main study and of this Sjogren substudy was to perform unsupervised clustering with no predefined outcomes. These studies were not built to assess the effect of any medical or drug intervention.

Flow Cytometry

Plots

Confirm that:

☒ The axis labels state the marker and fluorochrome used (e.g. CD4-FITC).

☒ The axis scales are clearly visible. Include numbers along axes only for bottom left plot of group (a 'group' is an analysis of identical markers).

☒ All plots are contour plots with outliers or pseudocolor plots.

☒ A numerical value for number of cells or percentage (with statistics) is provided.

Methodology

Sample preparation

Among the critical points reducing the reproducibility during flow cytometry studies are the instability of the fluorochromes conjugated to the antibodies (Abs) mainly of the tandem fluorochromes, and the pipetting errors of the reagents that may lead to changes in staining levels. To bypass these problems, we used Duraclone tubes (Beckman Coulter) specifically designed and optimized for the PRECISESADS study. These tubes correspond to ready-to-use unitized, dry format Ab cocktails. They eliminate errors due to manual Ab preparations, they improve the stability compared to liquid reagents, avoiding tandem breakdown, and are room temperature stable, thus excluding the need to manage varying expiration date and revalidations among single color liquid Abs.
Detailed standard operating procedures for sample staining with the Duraclone tubes have been already published in the Table 5 of our previous manuscript: (Jamin C, Le Lann L, Alvarez-Errico D, Barbarroja N, Cantaert T, Ducreux J, Dufour AM, Gerl V, Kniesch K, Neves E, Trombetta E, Alarcón-Riquelme M, Marañon C, Pers JO. Multi-center harmonization of flow cytometers in the context of the European "PRECISESADS" project. Autoimmun Rev. 2016 Nov;15(11):1038-1045. doi: 10.1016/j.autrev.2016.07.034. Epub 2016 Aug 1. PMID: 27490203).

Staining with panel 1 and panel 2:

1. Prepare the IOTest 3 lysing 1 ×/fixative solution (Beckman Coulter):
   a. Dilute the IOTest 3 Lysing Solution 10 × in distilled water.
   b. Add the IOTest 3 Fixative Solution according to the manufacturer's instructions.
   c. Mix well.

2. Drop by reverse pipetting the required volume of blood samples (either 50 µL or 100 µL) as indicated in the Standard Operating Procedure into the corresponding Duraclone tubes:
   a. Mix for 10 s to rehydrate the antibodies.
   b. Incubate the tubes for 20 min at room temperature in the dark.

3. Add 2 mL of the IOTest 3 lysing 1 ×/fixative solution at room temperature.
   a. Mix for 2 s.
   b. Incubate the tubes for 20 min at room temperature in the dark.

4. Add the FlowCount fluorospheres (Beckman Coulter).
   a. Mix the vial for 5 s and shake it 5 times by inversion.
   b. Add by reverse pipetting 50 µL or 100 µL of fluorosphres (same volume as blood with the same pipet).
   c. Mix for 2 s.

5. Tubes are ready for acquisition.
   a. Keep at 4 °C in the dark until acquisition.
   b. Acquire the cells within 2 h after preparation.

Instrument

Multi-parameter flow cytometry analyses have been performed in eleven different centers from the PRECISESADS consortium. Therefore, the integration of all data in common bioinformatical and biostatistical investigations has required a fine mirroring of all instruments [Le Lann L, Jouve PE, Alarcón-Riquelme M, Jamin C, Pers JO; PRECISESADS Flow Cytometry Study Group; PRECISESADS Clinical Consortium. Standardization procedure for flow cytometry data harmonization in prospective multicenter studies. Sci Rep. 2020 Jul 14;10(1):11567. doi: 10.1038/s41598-020-68468-3. PMID: 32665668; PMCID: PMC7360585]. The calibration procedure elaborated to achieve this prerequisite and the antibody panels used have been previously described [Jamin, C. et al. Multi-center harmonization of flow cytometers in the context of the European "PRECISESADS" project. Autoimmun Rev. 15, 1038-1045 (2016)]. In brief, In the eleven sites responsible for the flow cytometry acquisition, a Navios flow cytometer (Beckman Coulter) was used in three centers, a Gallios (Beckman Coulter) in one center, a FACS Canto II (BD Biosciences) in four centers and a FACS Aria III, a FACS Verse and a LSR Fortessa (BD Biosciences) in one center each. All instruments are equipped with three lasers emitting at 405/407, 488, and 633/635 nm and with optical filter configuration permitting the detection of FITC, PE, PC5.5, PC7, APC, APC-AF750, PB, and KO fluorochromes. VersaComp Ab Capture Bead kit (Beckman Coulter) is used for the photomultiplier tube (PMT) adjustments and the determination of the target MFI values applied for the multicenter harmonization procedure. Eight-peak Rainbow bead calibration particles (Spherotech) are utilized over the 5-year duration of the study for the daily checks as monocenter verification of the instrument stability. The same lots of VersaComp capture beads (#4,131,003 K) and of 8-peak Rainbow beads (#AF01) were ordered in all centers. If a new lot of beads needs to be ordered, the procedure described below will be repeated and the targets adjusted according to the PMT voltages fixed with the primary lot.

Software

The strategy developed to avoid any redundancy in the different cell subsets and to increase the accuracy of the phenotypes has been automated by AltraBio (Lyon, France) with a subcontract. The automation of gating has been built using a supervised Machine Learning-based approach using training datasets gated manually. This automation required a two steps workflow. The first step was customized for each flow cytometer due to potential strong differences in signal for the Forward Scatter and Side Scatter (FS / SS) measures across the different instruments used in the study. The second non instrument specific step was for gating all remaining populations of interest. In order to validate the Machine Learning based algorithms (name automatons), intermediate evaluations have been carried out. The results generated by the automatons were compared with manual gating analysis performed by the same operator with the Kaluza® software on 300 patients distributed throughout the centres=.. The comparison of the results showed a very good correlation of the data of frequencies, absolute values and the MFIs (coefficient of correlation 0.9996) making it possible to validate the efficiency of the automatons. This strategy has been published in the following manuscript [Le Lann L, Jouve PE, Alarcón-Riquelme M, Jamin C, Pers JO; PRECISESADS Flow Cytometry Study Group; PRECISESADS Clinical Consortium. Standardization procedure for flow cytometry data harmonization in prospective multicenter studies. Sci Rep. 2020 Jul 14;10(1):11567. doi: 10.1038/s41598-020-68468-3. PMID: 32665668; PMCID: PMC7360585].

Cell population abundance

No sort in this paper. The distribution of the different cell subset is provided in supplementary figure 6.

Gating strategy

All the gating strategy is described in the manuscript (Le Lann L, Jouve PE, Alarcón-Riquelme M, Jamin C, Pers JO; PRECISESADS Flow Cytometry Study Group; PRECISESADS Clinical Consortium. Standardization procedure for flow cytometry data harmonization in prospective multicenter studies. Sci Rep. 2020 Jul 14;10(1):11567. doi: 10.1038/s41598-020-68468-3. PMID: 32665668; PMCID: PMC7360585.) and available with the following link:
file:///C:/Users/JACQUE~1/AppData/Local/Temp/41598_2020_68468_MOESM6_ESM.pdf. This figure is now added in the supplementary material file.
The gating strategy was as follows: after exclusion of debris, dead cells and doublets, frequencies and absolute numbers of CD15hiCD16hi neutrophils, CD15hiCD16+ eosinophils, CD14+CD15hi LDGs, CD14hiCD16- classic monocytes, CD14+/hiCD16+ intermediate monocytes, CD14-CD16+ non classic monocytes, CD3+ T cells (with CD4+CD8-, CD4+CD8+, CD4-CD8+, CD4-CD8- T cell subsets), CD19+B cells, CD3-CD56+ NK cells (with CD16-CD56hi and CD16hiCD56lo NK cell subsets), CD3+CD56+ NK-like cells, Lin-HLA-DR+ DCs (with CD11c-CD123hi pDCs, CD11c+CD123lo mDCs (with CD141-CD1c+ mDC1, CD141+CD11c- mDC2 and CD141-CD1c- mDC subsets)) and CD123+HLA-DR- basophils were automatically extracted from FCS and LMD files of 283 patients and 309 HV and sent in an Excel flow cytometry workflow.

☒ Tick this box to confirm that a figure exemplifying the gating strategy is provided in the Supplementary Information.
